# Self-assembled monolayers of reduced graphene oxide for robust 3D-printed supercapacitors

**DOI:** 10.1038/s41598-024-65635-8

**Published:** 2024-07-01

**Authors:** Davide Scarpa, Mariagrazia Iuliano, Claudia Cirillo, Pierpaolo Iovane, Carmela Borriello, Sabrina Portofino, Eleonora Ponticorvo, Sergio Galvagno, Maria Sarno

**Affiliations:** 1 Department of Physics “E.R. Caianiello”, University of Salerno, Via Giovanni Paolo II, 132-84084 Fisciano, Italy; 2 NANO_MATES Research Centre, University of Salerno, Via Giovanni Paolo II, 132-84084 Fisciano, Italy; 3 Nanomaterials and Devices Laboratory (SSPT-PROMAS-NANO), ENEA, Italian National Agency for New Technologies, Energy and Sustainable Economic Development, Piazzale E. Fermi 1, 80055 Portici, NA Italy

**Keywords:** Reduced graphene oxide, Additive manufacturing, Self-assembled monolayers, Supercapacitors, Functionalized particles, Energy storage, Nanoscale materials

## Abstract

Herein, additive manufacturing, which is extremely promising in different sectors, has been adopted in the electrical energy storage field to fabricate efficient materials for supercapacitor applications. In particular, Al_2_O_3_-, steel-, and Cu-based microparticles have been used for the realization of 3D self-assembling materials covered with reduced graphene oxide to be processed through additive manufacturing. Functionalization of the particles with amino groups and a subsequent "self-assembly" step with graphene oxide, which was contextually partially reduced to rGO, was carried out. To further improve the electrical conductivity and AM processability, the composites were coated with a polyaniline-dodecylbenzene sulfonic acid complex and further blended with PLA. Afterward, they were extruded in the form of filaments, printed through the fused deposition modeling technique, and assembled into symmetrical solid-state devices. Electrochemical tests showed a maximum mass capacitance of 163 F/g, a maximum energy density of 15 Wh/Kg at 10 A/g, as well as good durability (85% capacitance retention within 5000 cycles) proving the effectiveness of the preparation and the efficiency of the as-manufactured composites.

## Introduction

In the last few years, Additive Manufacturing (AM), also known as either Rapid Prototyping (RP) or Solid Freeform Fabrication (SFF), is a term that has been more and more frequently used in the industrial field. Wherever there is a need for rapid prototyping for both aesthetic and/or practical purposes, in every production sector, AM is an effective solution. Described for the first time in 1986 by Charles Hull^1^, this process consists of a bottom-up method for manufacturing three-dimensional objects with high geometric precision starting from computerized 3D models, generally by either depositing or polymerizing the chosen material layer by layer with an unprecedented degree of freedom. This technology can bring many advantages over other ones, such as, for instance, subtractive manufacturing and injection molding. Firstly, owing to the layer-by-layer technology, it is possible to recreate almost any complex shape, including concave or protruding parts, of a wide range of materials for several applications. Moreover, whereas in subtractive manufacturing a quantity of waste material is generated (i.e. the initial block excluded part for obtaining the desired object), in additive manufacturing no wastes are generated, except a negligible amount of possible supports. This leads to a significant reduction of production costs, as well as of the costs related to disposal, transport, and environmental pollution, with a minimal adoption of hazardous chemicals (e.g. solvents for etching and cleaning). Furthermore, compared to classic production methods such as injection molding, AM guarantees extremely shorter times, especially in the case of rapid prototyping^2–5^.

On the other hand, electrical energy storage (EES) has recently stood out as one of the most scientifically and industrially relevant issues. Among storage systems, supercapacitors allow the fabrication and easy diffusion of efficient EES devices owing to their outstanding power density, rapid charge/discharge property, superior long-cycle life, and lower toxicity of the adopted materials^6–8^. Typically, manufacturing involves slurry preparation, coating, and drying steps to produce electrode films with limited thicknesses. These processes are energy and time-intensive and involve the handling of significant amounts of toxic solvents. In this regard, AM technologies represent a real turning point and development possibility in the field of electrochemistry, too. They provide a unique platform, when compared with other electrode manufacturing processes, such as photolithography and nanoarchitecture, thanks to their extreme versatility and adaptability. These technologies do not require base supports, different and complex steps to prepare electrochemical components and particularly specialized operators. The electrode, also with complex architectures, can be obtained extremely quickly in a single step^9,10^. Furthermore, research in this area has not yet achieved technological maturity^11–13^. More in general, the design and preparation of conductive composites, containing active materials, processable in 3D, is an interesting prospect even just for the intrinsic innovation to the field.

To find efficient supercapacitor electrodes, recent research has been focused on graphene-based materials^14^. In particular, reduced graphene oxide (rGO), obtained from the reduction of graphene oxide (GO), represents an excellent choice^15,16^. Indeed, it adds the advantages of the controlled density of oxygen-containing functionalities, enabling wettability with polar electrolytes as well as preventing aggregations of adjacent graphene sheets, to the remarkable capacitive behavior of high-surface-area and conductive graphene sheets^17^. rGO demonstrates superior conductivity, increased surface area, and enhanced stability at high voltages compared to GO^18^. Moreover, the presence of defects in rGO also contributes to a higher electrical permittivity than GO. The material's ability to sustain an electric field is considered a critical feature since it facilitates the formation and stability of the electrical double layer, thereby enhancing the overall capacitance performance^19,20^. Furthermore, the material structure, consisting of both oxygen-rich and oxygen-poor (graphitic) regions renders it an excellent candidate for a supercapacitor since the oxygen-rich regions between the graphitic regions can effectively favor charge carriers accumulation^21^. However, due to intrinsic internal mechanical fragilities, which could lead to reduced durability and electrochemical performance, creating a stable and robust 3D architecture incorporating rGO sheets is a concrete challenge to face. On the other hand, GO functionalities allow for spontaneous self-assembled monolayer (SAM) on suitable more robust functionalized materials^22–24^. In this process, GO can typically transform into its reduced form. Various driving forces have been explored in the literature to make assembly possible, such as hydrophilic/hydrophobic interactions, pH differences, etc. In general, by varying the functional groups of the underlying SAM, different interaction forces with the chosen assembly material can occur.

To obtain robust and efficient 3D supercapacitor electrodes, easily manufactured through AM, the choice of rGO support is fundamental. Among the materials adopted for AM, aluminum oxide, which, it is expected, does not hinder the capacitance performance of rGO, is commonly used, due to its versatility, low price, and relatively low sintering temperature^25^. It is also characterized by high strength, low friction coefficient, excellent wear and electrolytic environment corrosion resistance. Although exhibiting lower conductivity than other metal oxides, Al_2_O_3_ is widely utilized in catalysis because of its high chemical stability and large specific surface area^26^. It has been reported that aluminum oxide, when incorporated into composites for supercapacitor applications, not only enhances capacitance by enlarging the contact area between the composite and the electrolyte solution^27^, but also prevents the collapse of the composite in the electrolyte, thereby improving the cycling stability of the device^28–30^.

Steel is another material of great interest, due to several advantages such as: (i) good corrosion resistance; (ii) ductility, hardness, toughness, and wear resistance; (iii) low cost; (iv) variety of microstructural characteristics, ranging from ultra-hard martensite to multiphase compounds. Steel mesh is also frequently adopted as a component of supercapacitor electrodes^31^. Thus, when incorporated into a supercapacitor, steel can impart specific mechanical properties such as enhanced lightweight and flexibility. Additionally, it serves as an appealing conducting substrate due to its affordability and ease of processing^32^. Moreover, when utilized in microparticle form, it offers the added benefit of a larger surface area compared to flat steel sheets, thereby enhancing capacitance^33^.

Furthermore, copper is a transition metal that has also drawn great attention, also, in the AM field. Copper is a ductile metal, characterized by good corrosion resistance, low chemical reactivity, extraordinary processability as well as high electrical conductivity (around 60 × 10^6^ S/m)^34^. Owing to its unique characteristics, copper is frequently adopted in devices used in various applications: in electronics, in the manufacture of radiators, intercoolers and heat exchangers, in electrochemistry either as the substrate or combined with other materials^35^. Additionally, several copper architectures have been also widely adopted in the field of supercapacitors, either as low-resistance current collector^36^ or active electrode material, distinguished by the presence of copper or copper oxide^37–40^ as well as copper compounds^41^. In the implementation of AM technologies, although the opportunity to obtain high-performance components stimulates extensive scientific research^42^, the processability of copper and copper alloys faces several challenges due to high electrical and thermal conductivity and tendency to oxidation^43^.

With the aim of exploring a versatile and adaptable approach giving robust and performing 3D printed supercapacitors, various architectures, that benefit from graphene covering, were reported and studied in the following. In the present study, Al_2_O_3_-, steel-, and Cu-based microparticles have been explored for the realization of 3D self-assembling materials covered with rGO to be processed through AM. To favor GO "self-assembly" a functionalization of the particles with amino groups was designed. During this process, GO was contextually partially reduced to rGO. Furthermore, the electrical conductivity and AM processability were improved by covering with a complex consisting of dodecylbenzenesulfonic acid-functionalized polyaniline and finally easily AM processable polylactic acid was blended. Subsequently, they were extruded in the form of filaments to be printed through Fused Deposition Modeling (FDM) in circular disc electrodes which were, eventually, assembled in solid-state electrolyte-based symmetric devices. Finally, the electrochemical performance was analyzed using cyclic voltammetry (CV) and galvanostatic charge–discharge (GCD) curves.

## Materials and methods

### Materials preparation

**Figure 1:**
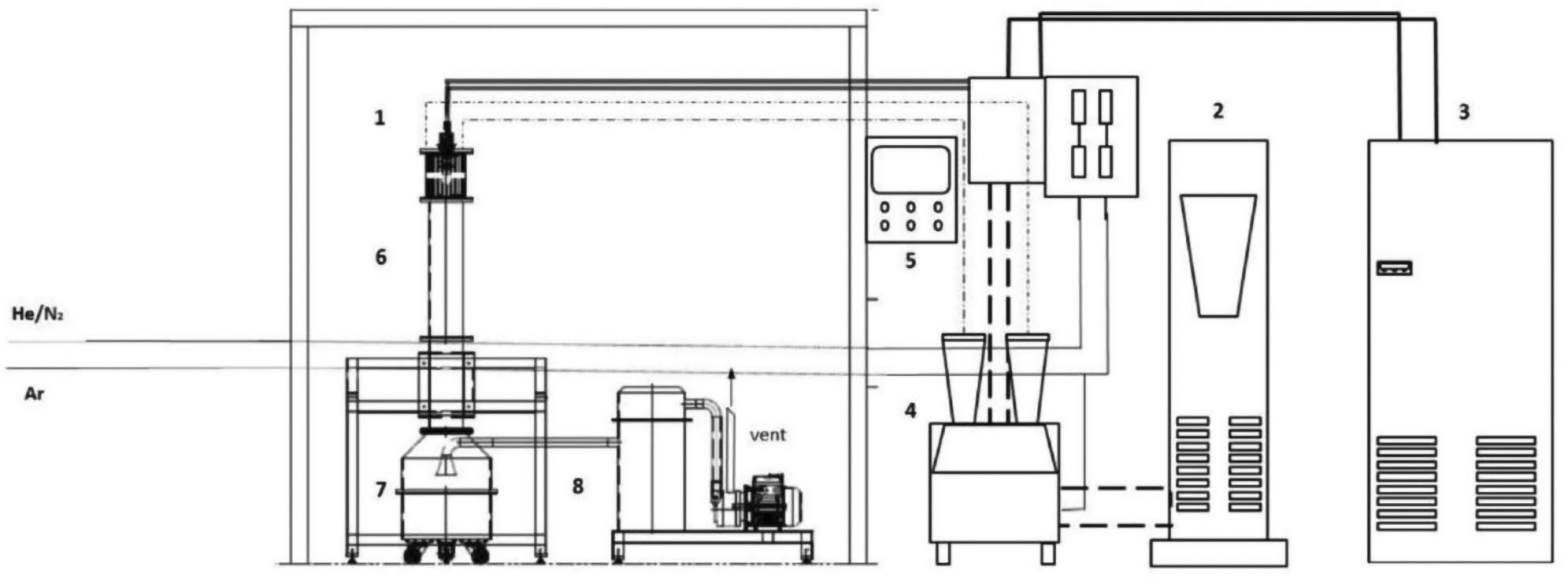
Scheme of the DC non transferred arc plasma plant: (1) Plasma torch; (2) Power supply; (3) Quencher; (4) Powder feeder; (5) Control unit, (6) Reactor; (7) Collection tank; (8) Bag filter.

Al_2_O_3_ and steel microparticles were obtained via thermal plasma synthesis from commercial Al_2_O_3_ powder (Sigma-Aldrich) and commercial steel powder (Sigma-Aldrich). In particular, the samples were produced in a pre-pilot plant, which was designed and installed at the ENEA-Portici Research Centre and built by Praxair surface technologies^44^. A scheme of the plant, based on thermal plasma technology, is reported in Fig. 1. The plasma system is equipped with a DC non-transferred torch with a maximum power of 40 kW, a power supply of 80 kW, a dry scroll vacuum pump, as well as a bag filter. The torch is located on the top of a cold water-cooled jacketed-cylindrical stainless-steel reactor and the system works under a light vacuum (60–20 mbar). Processed powders are collected in a tank located below the reactor. During reactions, the above-mentioned powders, continuously fed to the reactor using a pneumatic feeding system, are sprayed through a nozzle located at the top of the reactor, horizontally with respect to the plasma flame. Tests were carried out adopting argon (Ar) as the main gas to light up the plasma, whereas helium (He) was selected as a secondary gas to enhance the flame conditions. Within the reactor, the powders underwent an evaporation/reconditioning reaction in a few milliseconds of residence time and then were dragged out by the process gas. The rapid cooling beyond the reaction zone limited the growth of the particles. As for Cu particles, they were commercial particles purchased from Sigma-Aldrich.

### Coating and functionalization of particles

In view of the production through additive manufacturing of the supercapacitor devices, the above-described particles underwent a functionalization process which can be summarized in different steps.

#### First and second steps: functionalization of particles and their coating with rGO

The particle surface was first functionalized with amino groups and then linked to graphene oxide, which covered the functionalized particles in the form of reduced graphene oxide.

**Figure 2:**
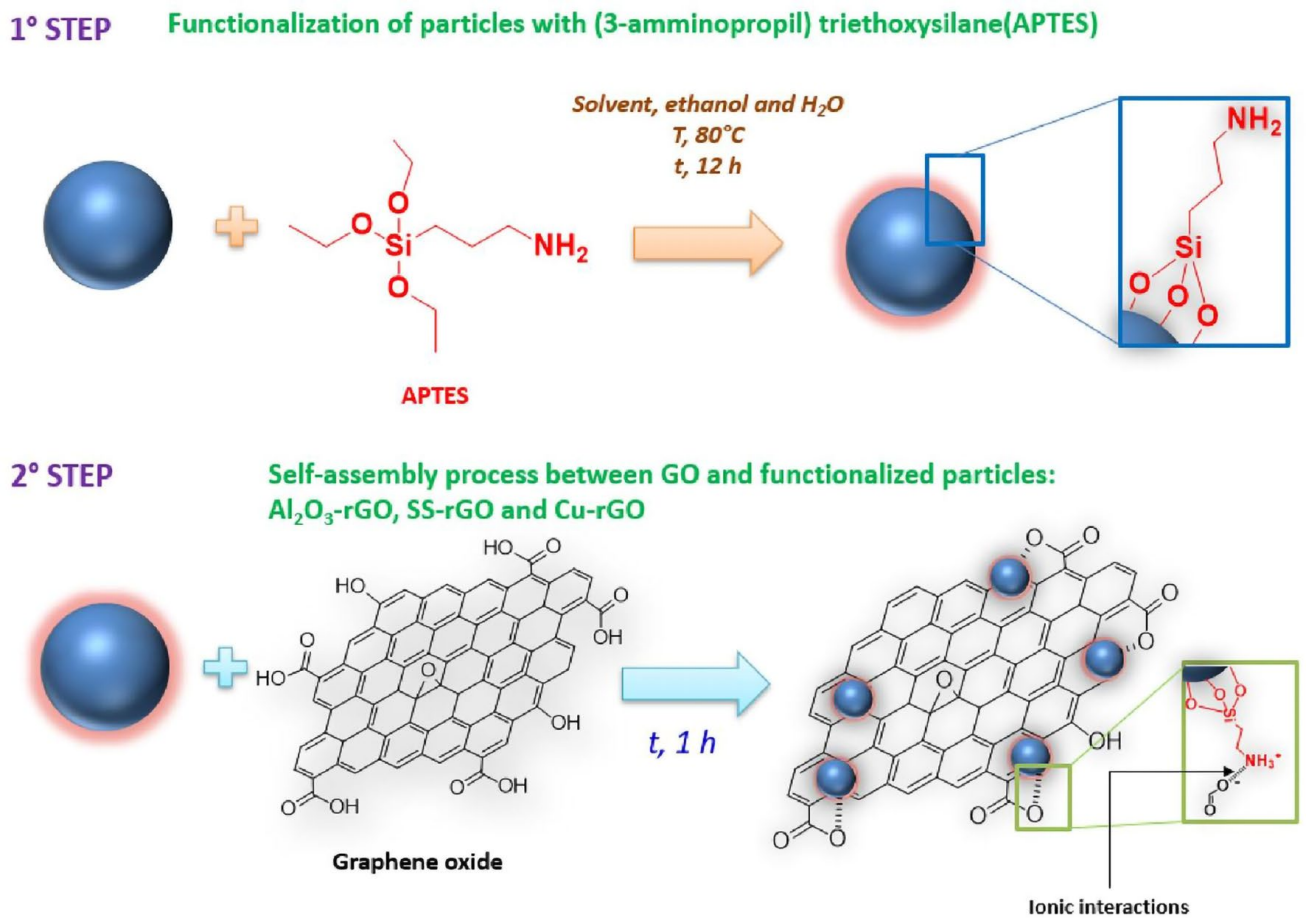
Scheme of the two-step process of coating functionalized particles with rGO.

**Figure 3:**
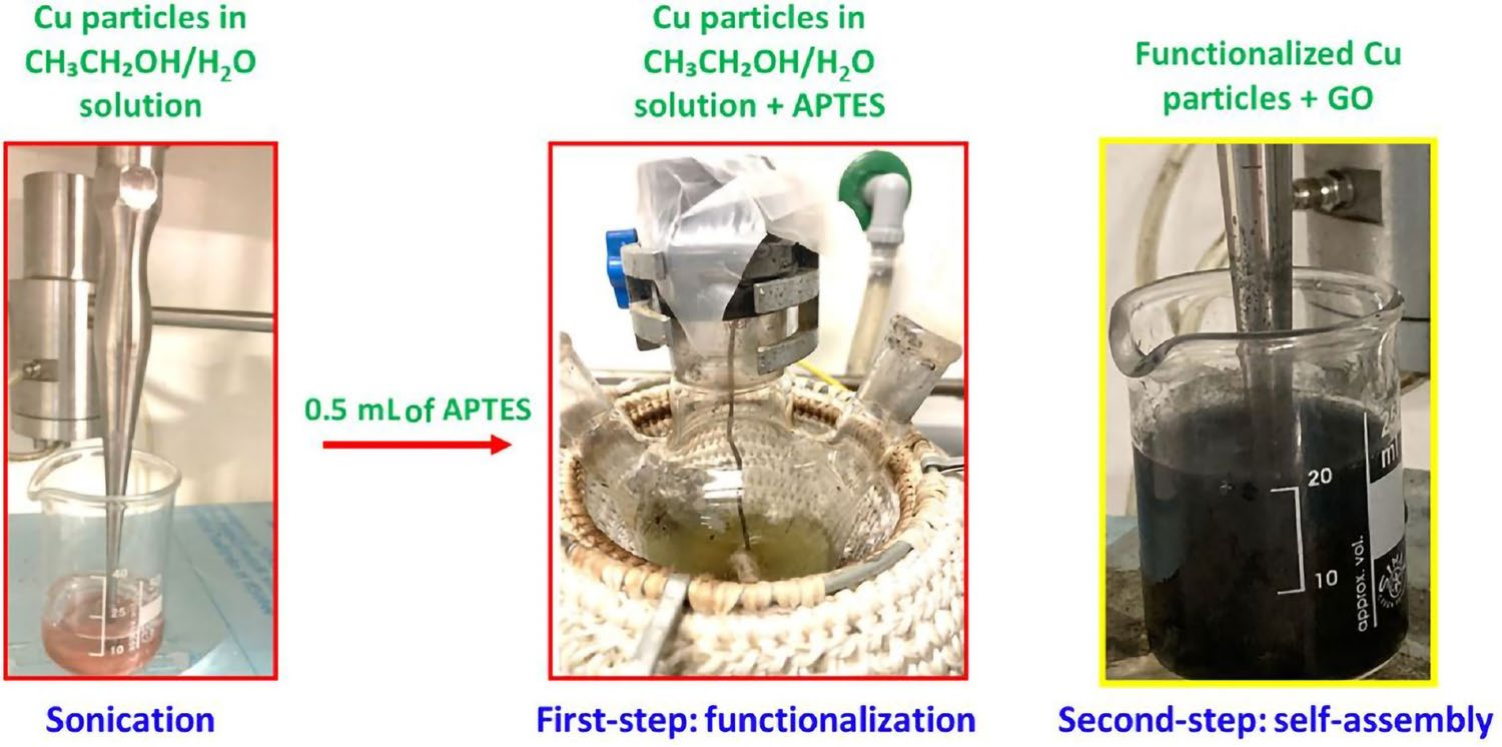
Photos of the two-step process of coating for functionalized Cu particles with rGO.

In detail, rGO-coated particles were prepared by means of a two-step process, as represented in Fig. 2, whereas photos of the process related to Cu particles are reported in Fig. 3.

The first step involved functionalization of the particles with (3-aminopropyl) triethoxysilane (APTES). In detail, 200 mg of particles were dispersed in a solution containing 10 mL of ethanol and 5 mL of deionized water. Afterward, the solution was sonicated for a few minutes before adding 0.5 mL of APTES to it. A functionalization step was then carried out at 80 °C for 12 h under magnetic stirring; after this procedure, the products were centrifuged several times in ethanol to obtain particles functionalized with amino groups. This functionalization made the particles positively charged^45^.

The second step involved a self-assembly process between GO and the amino-functionalized particles. In particular, 10 mg of GO were added to 20 mL of water and the pH of the solution was turned into the range 4–6 through the addition of diluted sodium hydroxide. At the same time, a second mixture was prepared by adding 200 mg of amino-functionalized particles to 20 mL of deionized water, and the pH of this suspension was adjusted to 3 by adding a diluted hydrochloric acid solution. Eventually, under magnetic stirring, the GO-containing solution was added dropwise to the amino-functionalized particles-containing suspension. A 1-h sonication step of the as-obtained solution followed. During this phase, the negatively charged graphene oxide, after the ionization of its functional groups (such as carboxylic groups) has occurred in solution, underwent self-assembly with the particles owing to electrostatic interactions generated between the above-mentioned negative charges and the positively charged particles through their superficial amino groups; contextually, GO was partially reduced to rGO.

#### Third step: synthesis of the PANI-DBSA complex

**Figure 4:**
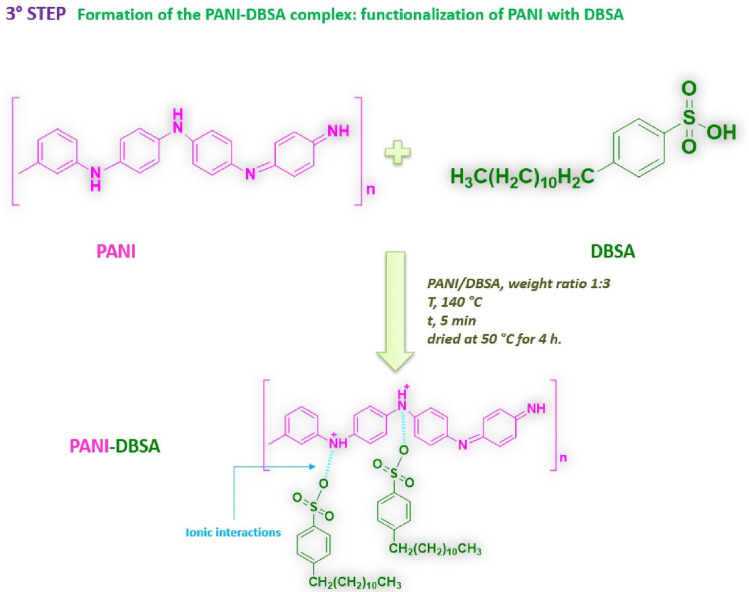
Scheme of the synthesis of the PANI-DBSA complex.

The third step consisted of the synthesis of the polyaniline-dodecylbenzene sulfonic acid (PANI-DBSA) complex (see Fig. 4). PANI is a polymer characterized by high electrical conductivity, being therefore adopted to produce several devices such as supercapacitors, photoelectric devices, sensors, etc. Unfortunately, conductive polymers are not compatible with additive manufacturing^46^ due to their poor processability and their low thermal stability. In fact, at high temperatures, PANI decomposes, hence losing its conductive nature. For this reason, PANI, before being anchored on the functionalized particles, was functionalized with the DBSA molecule to ensure high conductivity at the processing temperatures necessary for 3D printing. In particular, PANI (emeraldine base) was mixed with DBSA at 140 °C for 5 min with a PANI/DBSA weight ratio equal to 1:3, and the as-prepared solution was subsequently dried at 50 °C for 4 h.

#### Fourth step: functionalization of the rGO-coated particles with the PANI-DBSA complex

**Figure 5:**
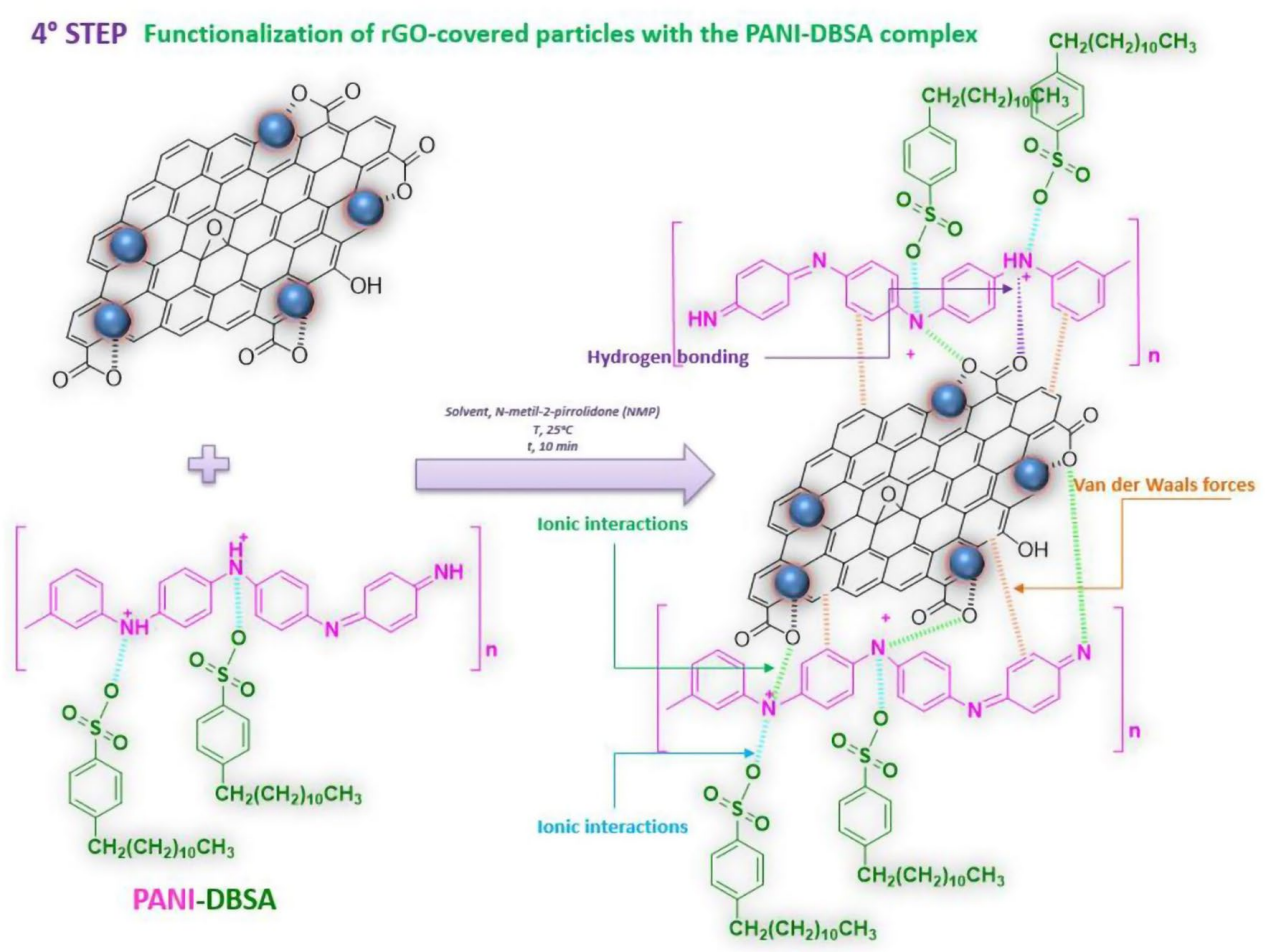
Scheme of functionalization of the rGO-coated particles with the PANI-DBSA complex.

The fourth step consisted of a self-assembly process between the functionalized particles coated with rGO and the solution containing the PANI-DBSA complex, as summarized in Fig. 5. Once the PANI-DBSA complex was obtained, it was dispersed into N-methyl-2-pyrrolidone to obtain a 30 mg/mL solution. The as-obtained solution was then added to 6 mL of a 16 mg/mL dispersion of the rGO-coated particles. The mixture was then stirred for 10 min at room temperature to obtain a composite consisting of the rGO-coated particles functionalized with PANI-DBSA. The three composites obtained will be named as follows: Steel-rGO@PANI-DBSA, Al_2_O_3_-rGO@PANI-DBSA, and Cu-rGO@PANI-DBSA.

### Blending with PLA, 3D printing and preparation of devices

In recent decades, the growing demand for polymeric materials capable of ensuring new and fascinating properties as well as providing high performance has pushed research forward into the creation of blends obtained by mixing different polymers. The main reason for this type of development lies in the possibility of obtaining a final product whose properties can be appropriately tuned depending on the type of components and how they are mixed. This allows us to respond more quickly to market needs, significantly reducing the time and investment required for the identification of new innovative polymers. Furthermore, blending also offers advantages in terms of improved processability and product homogeneity. For the above-mentioned reasons, the blending process was taken into consideration to improve the processability of PANI at high temperatures^47^. In particular, the amino-functionalized particles covered with rGO, and further functionalized with the PANI-DBSA complex were mixed with a thermoplastic polymer, polylactic acid (PLA). PLA is the most widely used biodegradable thermoplastic aliphatic polyester, as it is readily available, biocompatible and shows a rather good mechanical strength. Owing to its low cost, its low melting temperature and its minimal warping, PLA is one of the easiest materials to be successfully adopted in AM, especially in FDM^48–50^. Therefore, Steel-rGO@PANI-DBSA, Al_2_O_3_-rGO@PANI-DBSA and Cu-rGO@PANI-DBSA were mixed with commercial PLA (Sigma Aldrich) in order to obtain a much more AM processable material. In this procedure, composites were blended with PLA at a weight ratio of 1:2 (composites to PLA), which was the optimized ratio to ensure the best 3D printing processability. The mixture was heated to 200 °C and thoroughly mixed until a uniform compound was achieved. Subsequently, the compound was cooled down to room temperature.

**Figure 6:**
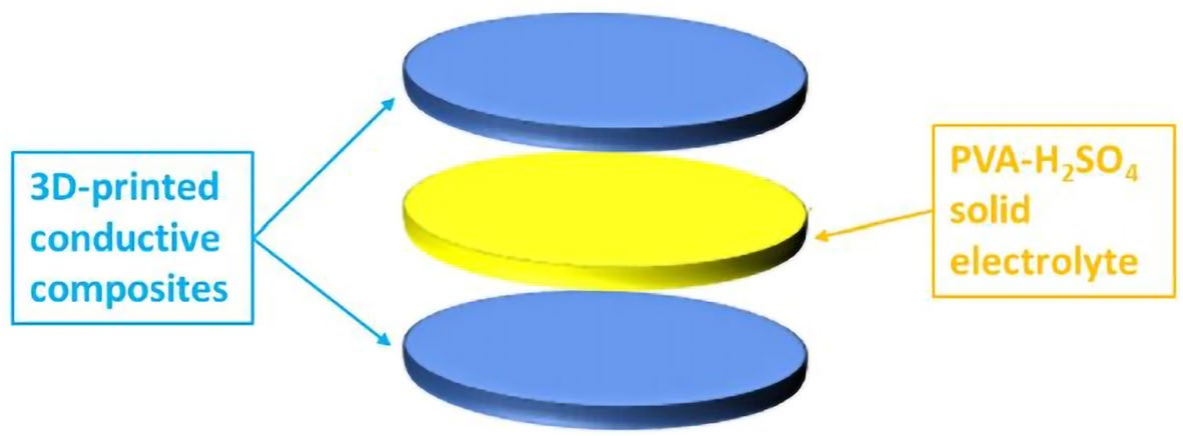
Schematic representation of a supercapacitor device.

The resulting PLA-loaded composites were then placed within a MiniCTW twin-screw extruder (ThermoScientifc) at a temperature of 200 °C and a screw speed of 30 rpm, obtaining a homogenous filament with a diameter of 1.75 mm. The 3D-printed model was designed via the CAD software Solidworks, and the corresponding gcode files were obtained through PrusaSlicer and eventually printed through a FDM technique using a Prusa i3 MK3S + 3D-printer (Prusa) to create a circular disc electrode with a diameter of 1 cm and a thickness of 2 mm. After printing the conductive composites in the form of disks, devices were built with the purpose of creating symmetrical supercapacitors. In particular, each device is composed of two 3D-printed discs, with a solid electrolyte inserted between the two discs to create a sandwich-like compact structure, as schematized in Fig. 6. The electrolyte was prepared by mixing 6 g of polyvinyl acetate (PVA) with 10 ml of a 1 M H_2_SO_4_ solution. The as-prepared symmetrical supercapacitors were named as steel-rGO@PANI-DBSA-PLA, Al_2_O_3_-rGO @PANI-DBSA-PLA and Cu-rGO@PANI-DBSA-PLA.

### Characterization techniques

Scanning electron microscopy (SEM) images were acquired through a LEO 1525 electron microscope (TESCAN), equipped with an energy-dispersive X-ray (EDX) probe. Powder X-ray diffraction (XRD) patterns were obtained with a Bruker D8 X-ray diffractometer using CuKα radiation. Thermogravimetry and derivative thermogravimetry (TG-DTG) were performed through an SDTQ 600 Analyzer (TA Instruments) with a 10 °C/min heating rate under air flow from room temperature to 800–900 °C.

Moreover, Fourier transform infrared (FT-IR) analysis (Vertex 70apparatus, Bruker Corporation) was performed. Thermo-Scientific Nicolet (i550 FT-IR) was used for Diffuse Reflectance Infrared Fourier Transform spectroscopy (DRIFTs) measurements.

Electrochemical measurements on composites before being mixed with PLA were obtained in a 0.5 M H_2_SO_4_ electrolytic solution. Before measurements, 8 mg of the synthesized samples were dispersed into 160 μl of a 5 wt% Nafion solution, 900 μl of 2-propanol, and 100 μL of water to obtain a homogeneous ink which, after a 30-min sonication and subsequent air-drying, was partially deposited dropwise onto a DRP-110 Screen Printed Electrode (SPE) made up of a carbon working electrode, a platinum counter electrode, and a silver reference electrode. SPEs were chosen due to their better properties over common carbon electrodes^51^.

Electrochemical measurements on both composites without PLA and printed devices were carried out using an Autolab PGSTAT302N potentiostat (Metrohm, Herisau, Switzerland).

The mass capacitance (*Csp*) of the devices has been obtained from galvanostatic charge–discharge curves according to the following formula^52,53^:

1 $$Csp=\frac{i\cdot \Delta \text{t}}{m\cdot \Delta \text{V}}$$

where *i* is the GCD current, *Δt* is the discharging time, *ΔV* is the potential window, and *m* is the catalyst loading mass.

Furthermore, the energy density (*E*) and the power density (*P* ) of each device have been evaluated according to the following equations:

2 $$E=\frac{1}{2}Csp\cdot {\Delta \text{V}}^{2}$$

3 $$P=\frac{E}{\Delta t}$$

where *Csp* is the mass capacitance calculated in (1), *Δt* is the discharging time and *ΔV* is the potential window.

## Results and discussion

### Characterization of the raw materials

#### Characterization of Al_2_O_3_ particles

**Figure 7:**
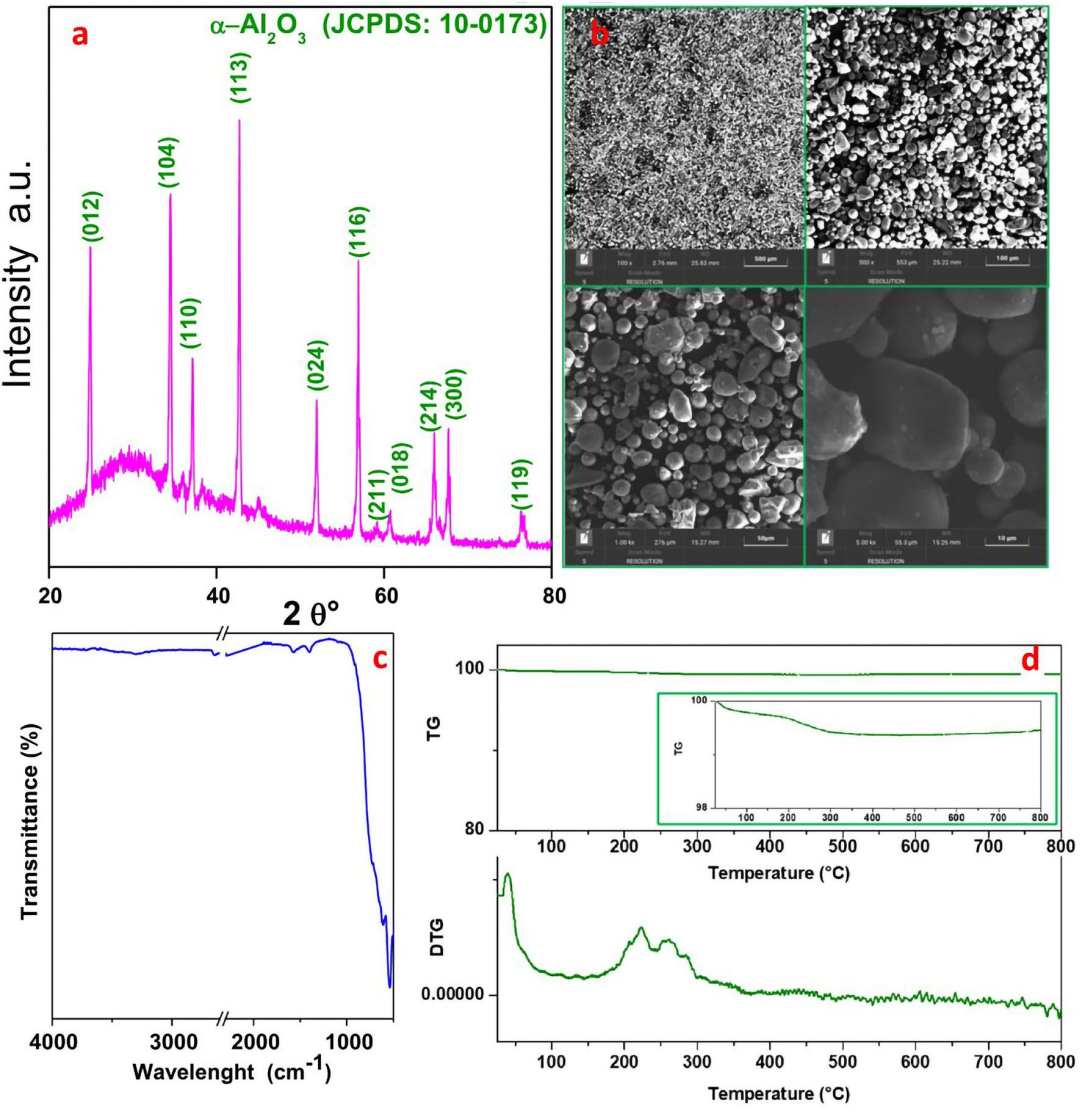
(**a**) X-ray diffraction pattern, (**b**) SEM images at different magnifications (scale bars: 500 μm, 100 μm, 50 μm and 10 μm), (**c**) FT-IR and (**d**) TG-DTG graphs under air-flow of Al2O3 particles.

The Al_2_O_3_ particles obtained through thermal plasma synthesis were first characterized through XRD analysis. As shown in Fig. 7a, the X-ray powder pattern confirms the presence of the α- Al_2_O_3_ phase of these particles, with peaks set respectively at values ​​of the angle 2θ equal to 24.80°, 34.55°, 36.89°, 43.90°, 51.49°, 56.87°, 59.32°, 60.67°, 65.80°, 67.89°, and 76.47°, corresponding to its typical (012), (104), (110), (113), (024), (116), (211), (018), (214), (300) and (119) crystal planes (JCPDS No. 10–0173).

Furthermore, SEM analysis allowed us to better understand the morphology of the sample which, as can be seen from Fig. 7b, is made up of rounded particles with average sizes in the range 5–150 µm. The measured BET surface area of the particles is 9.8 m^2^/g, the total pore volume is 0.014 cm^3^/g with a micropore volume of 0.002 cm^3^/g.

The FT-IR of the α-Al_2_O_3_ sample is shown in Fig. 7c: the IR profile shows a weak 3297 cm^−1^ band, characteristic of the stretching vibration of the -OH group linked to Al^3+54^. A vibrational band is also visible at 1574 cm^−1^, corresponding to the physisorbed water. At 1408 cm^−1^ the characteristic band of water deformation vibrations can be detected^55^. Finally, between 1000 cm^−1^ and 500 cm^−1^, bands due to the vibrational frequencies of the O–Al–O bonds are present^56,57^.

Furthermore, the thermogravimetric analysis of the alumina particles is shown in Fig. 7d. The thermogravimetric profile obtained under airflow shows a slight weight loss (as can be seen from the magnification shown in Fig. 7d in the green box) and the absence of significant decreasing steps, suggesting the presence of a single crystal phase and the absence of impurities. Weight loss (~ 1%) occurs at temperatures below 400 °C and is due to the evaporation of volatile components, such as water residues, including adsorbed water, free water, and crystalline water.

#### Characterization of steel particles

**Figure 8:**
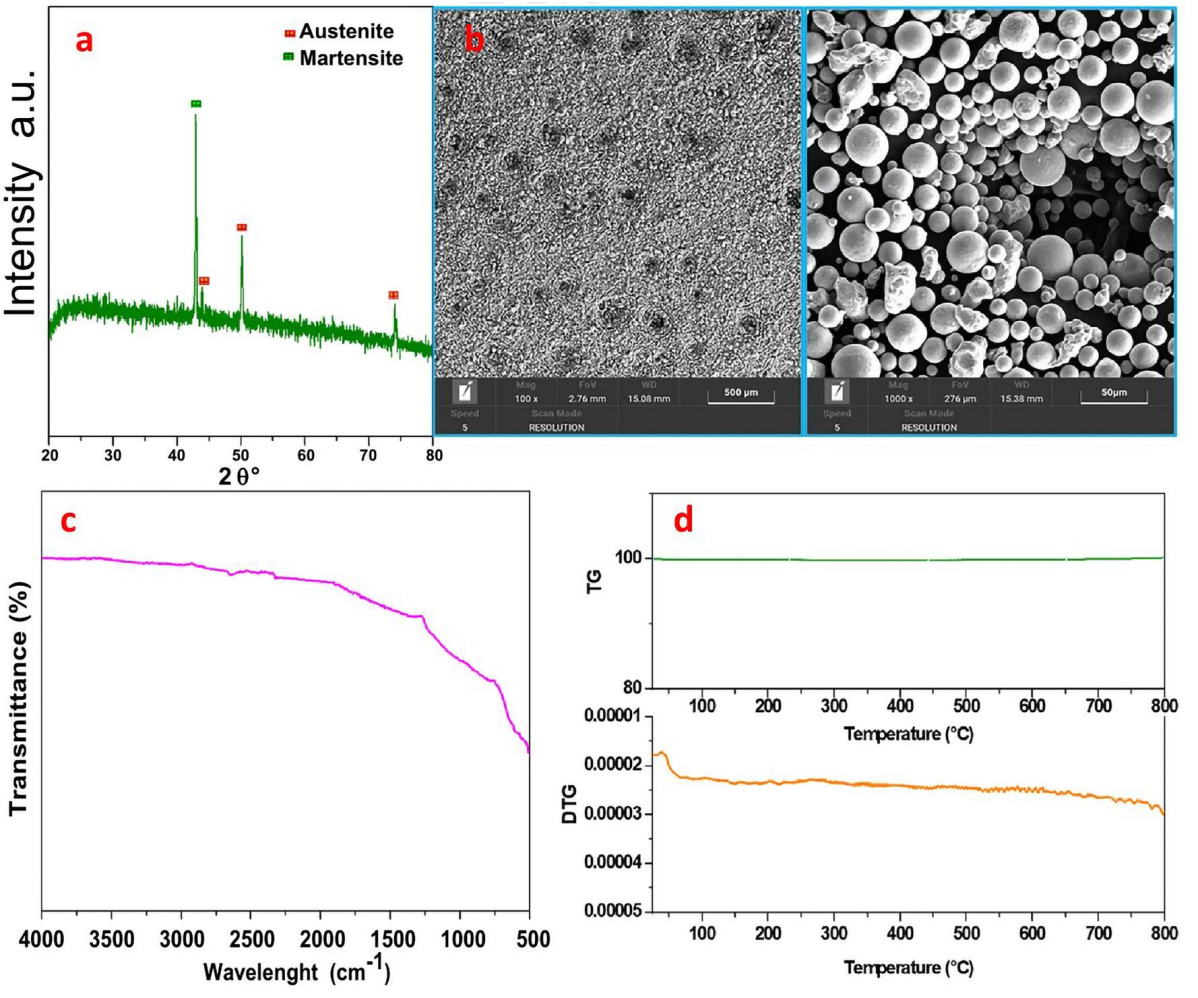
(**a**) X-ray diffraction pattern, (**b**) SEM images at different magnifications (scale bars: 500 μm, 50 μm), (**c**) FT-IR and (**d**) TG-DTG graphs under airflow of steel particles.

Figure 8a shows the X-ray powder pattern of the steel particles obtained through thermal plasma synthesis in the range of the 2θ angle between 20° and 80°. In the graph, the main peak related to the martensitic phase can be easily recognized at 42.86°^58^, whereas the peaks related to the crystal planes of the austenitic phase can be identified at 44.2°, 50.38° and 64.4°^59^.

Figure 8b shows the SEM images at different scale bars ranging from 500 μm to 50 μm. These images show particles with a quasi-spherical morphology and a size distribution in the range of 2–40 μm. The powder exhibits a BET surface area of 10.6 m^2^/g, total pore volume 0.021 cm^3^/g.

Figure 8c and d show, respectively, the FT-IR spectrum and the TG-DTG graphs of the steel particles. The FT-IR spectrum shows no bands attributable to organic components and there are no significant weight losses in the thermogram, which confirms the purity of the steel sample.

#### Characterization of Cu particles

**Figure 9:**
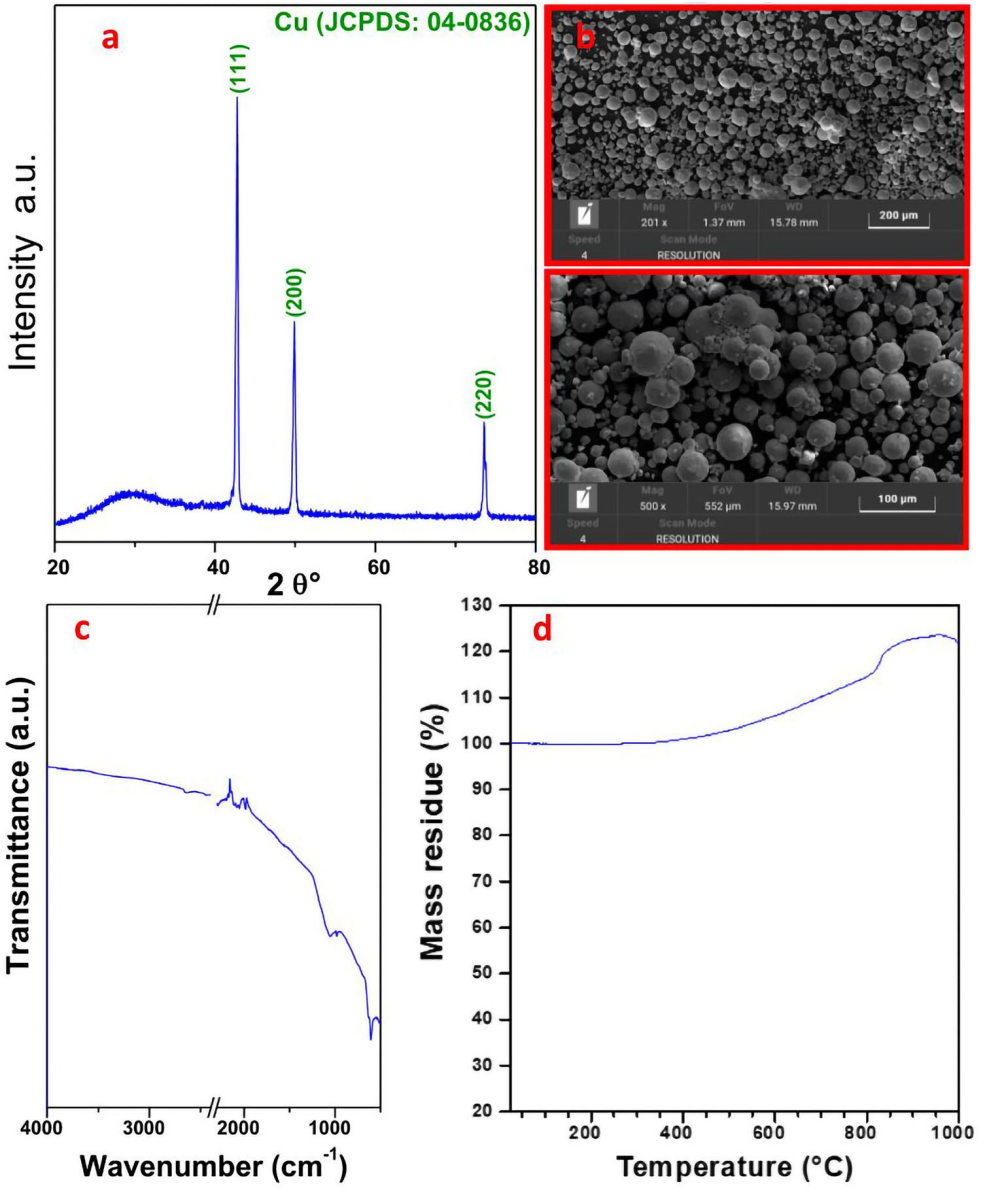
(**a**) X-ray diffraction pattern, (**b**) SEM images at different magnifications (scale bars: 200 μm and 100 μm) (**c**) FT-IR and (d) TG graph under airflow of Cu particles.

The copper particles were initially characterized through XRD analysis. The XRD diffraction pattern of the sample is shown in Fig. 9a: in particular, three distinct peaks can be identified at 2θ angle values ​​equal to 42.81°, 49.98°, and 73.63°, corresponding to the (111), (200) and (220) crystal planes of metallic Cu^60^. From the analysis of the spectrum, it can be stated that the sample has good crystallinity due to the sharpness of its major peaks.

Furthermore, the Cu powder was characterized through scanning electron microscopy. The SEM images of the sample (reported in Fig. 9b) show particles with a spherical morphology with sizes from 1 to 30 μm, the most centered at 8 μm. The powder BET surface area is 18,4 m^2^/g, total pore volume is 0.062 cm^3^/g.

Moreover, the analysis of the copper particles through FT-IR shows an absorption band at 620 cm^−1^, due to the vibration of the Cu–O bond of CuO^61^ (Fig. 9c) and an absorption band at approximately 1056 cm^−1^, which can be attributed to the C–OH stretching and to the OH bending vibration.

A thermogravimetric analysis under airflow carried out from room temperature to 1000 °C was also performed (Fig. 9d). The TG curve recorded an increase in the sample weight starting from the temperature of around 320 °C, attributed to the oxidation of the material which undergoes a percentage increase of about 25 wt% till 1000 °C.

The analysis of the rGO-coated particles evidences the occurrence of graphene oxide reduction (Figure S1, S2), probably due to its anchoring to the active sites of the amino-functionalized particles. Thermogravimetric analysis, shown in Figures S3, S4 and S5, shows weight losses in the temperature range of 200–700 °C. These losses can be attributed to the degradation of oxygenated functional groups and the degradation of C–C bonds in rGO. The weight losses vary among the three samples, with approximately 30%, 49%, and 70% for Al_2_O3, steel, and Cu particles, respectively. These discrepancies are likely due to differences in the particle size distribution, where Cu particles exhibit a more homogeneous and smaller size distribution, while Al_2_O_3_ particles have less circular larger particles.

### Characterization of PANI-DBSA complex

**Figure 10:**
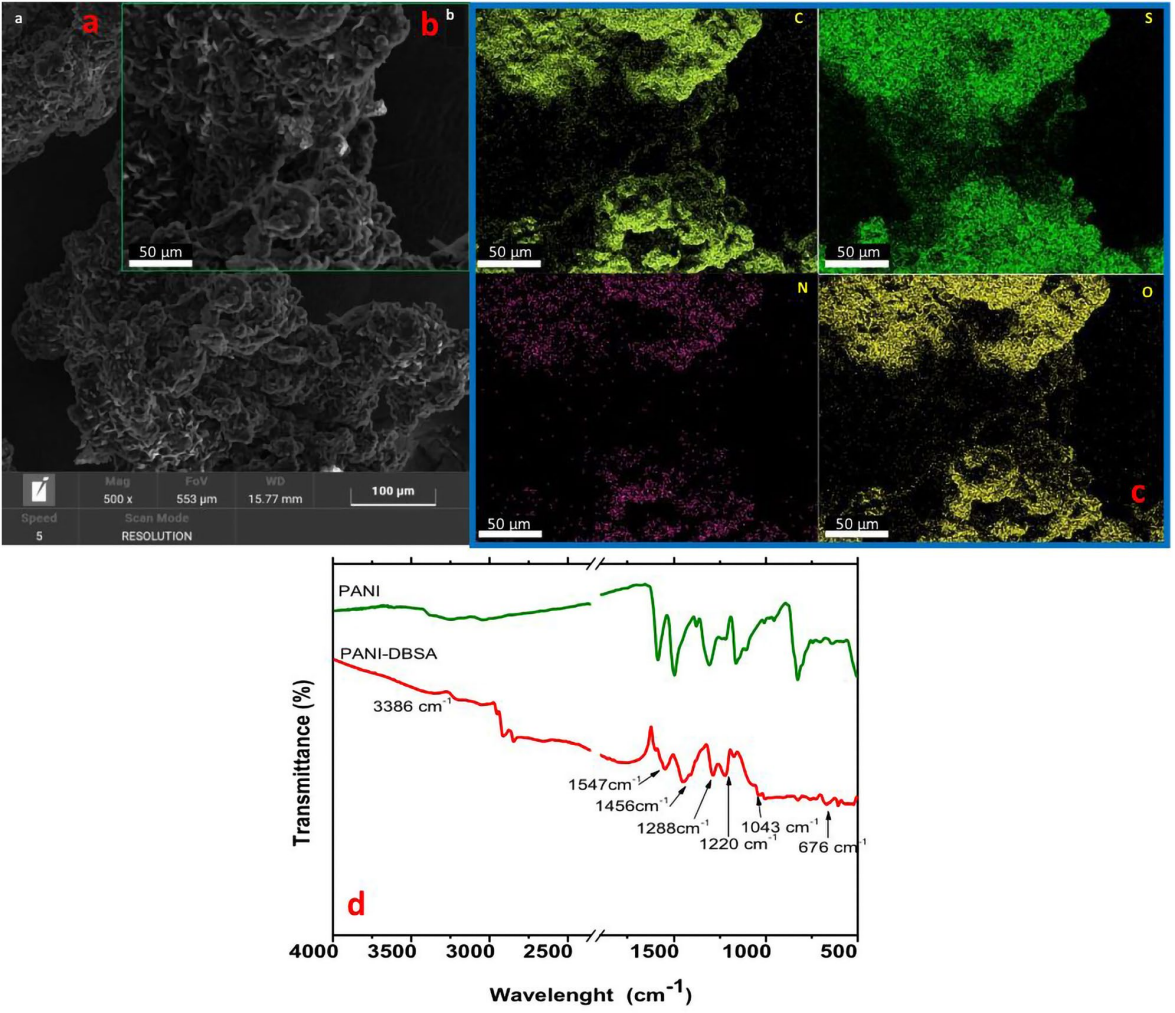
SEM images with scale bars of (**a**) 100 and (**b**) 50 µm; and (**c**) corresponding EDX maps of the PANI-DBSA complex. (**d**) FT-IR spectra of PANI (green profile) and PANI-DBSA complex (red profile).

Figure 10a shows an SEM image of the PANI-DBSA complex, while Fig. 10b reports an SEM image of the same sample at a higher magnification with the corresponding four EDX maps reported in Fig. 10c. The EDX maps highlight the presence of the C, N, O and S elements, thereby successfully confirming the functionalization of the complex. Figure 10d shows the FT-IR spectra of PANI and the DBSA-modified PANI in the range 500–3500 cm^−1^. The synthesized PANI-DBSA complex (red spectrum) shows a slight vibrational band related to the presence of the N–H bond at 3386 cm^−1^. There are also two absorption bands at 2923 cm^−1^ and 2853 cm^−1^, due respectively to the –CH_2_ and –CH_3_ bonds, generated by the aliphatic component of DBSA^62^. Furthermore, from the FT-IR spectrum of the PANI-DBSA composite, the characteristic bands of PANI are also visible at 1547 cm^−1^ and 1456 cm^−1^, representative of the C = C bond of the aromatic rings (quinoids and benzenoids, respectively). The positions of the bands are slightly shifted with respect to the ones in the spectrum of PANI alone (green spectrum), suggesting the successful DBSA doping into the PANI structure^63^.

The absorption bands observed at 1288 cm^−1^ and 1220 cm^−1^ correspond to the vibration of the C − N bond of the secondary aromatic amines of PANI^64^, while the band at 1043 cm^−1^ is due to the R − SO − bond of DBSA, and the band at 673 cm^−1^ corresponds to the stretching of the S = O bond of DBSA.

### Characterization of Al_2_O_3_-rGO@PANI-DBSA, steel-rGO@PANI-DBSA and Cu-rGO@PANI-DBSA composites

**Figure 11:**
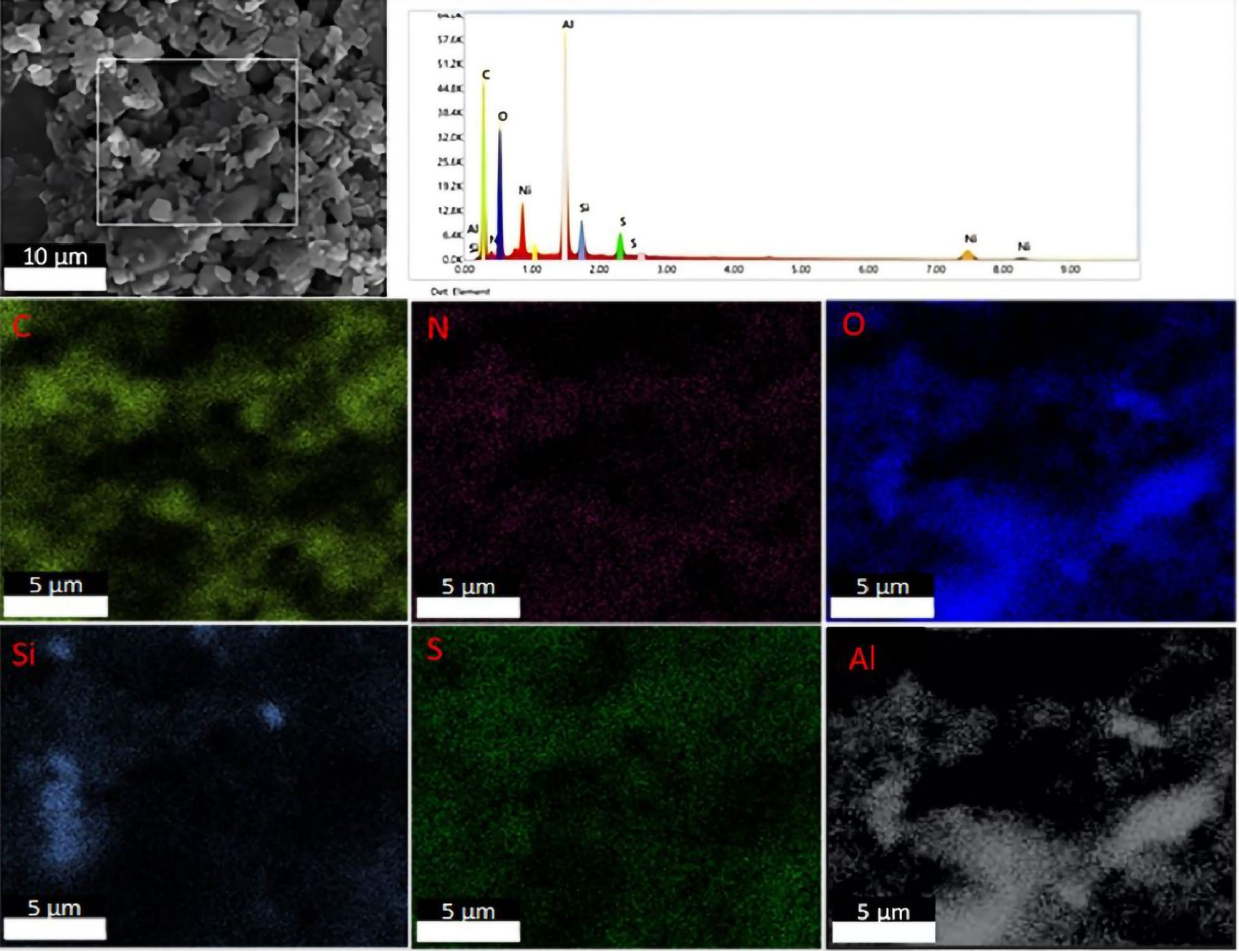
SEM–EDX analyses of Al2O3-rGO@PANI-DBSA.

**Figure 12:**
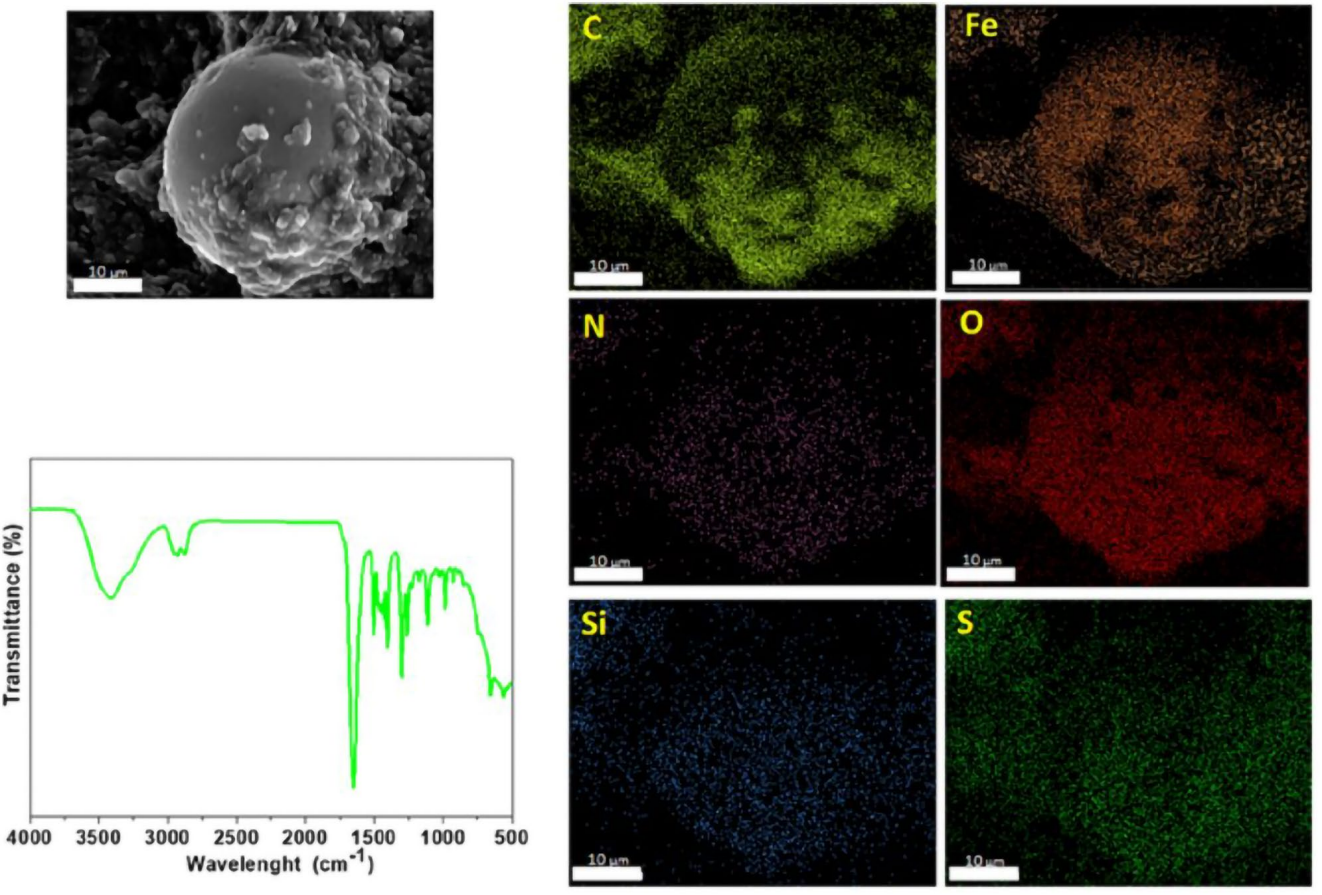
SEM–EDX analyses and FT-IR spectrum of steel-rGO@PANI-DBSA.

**Figure 13:**
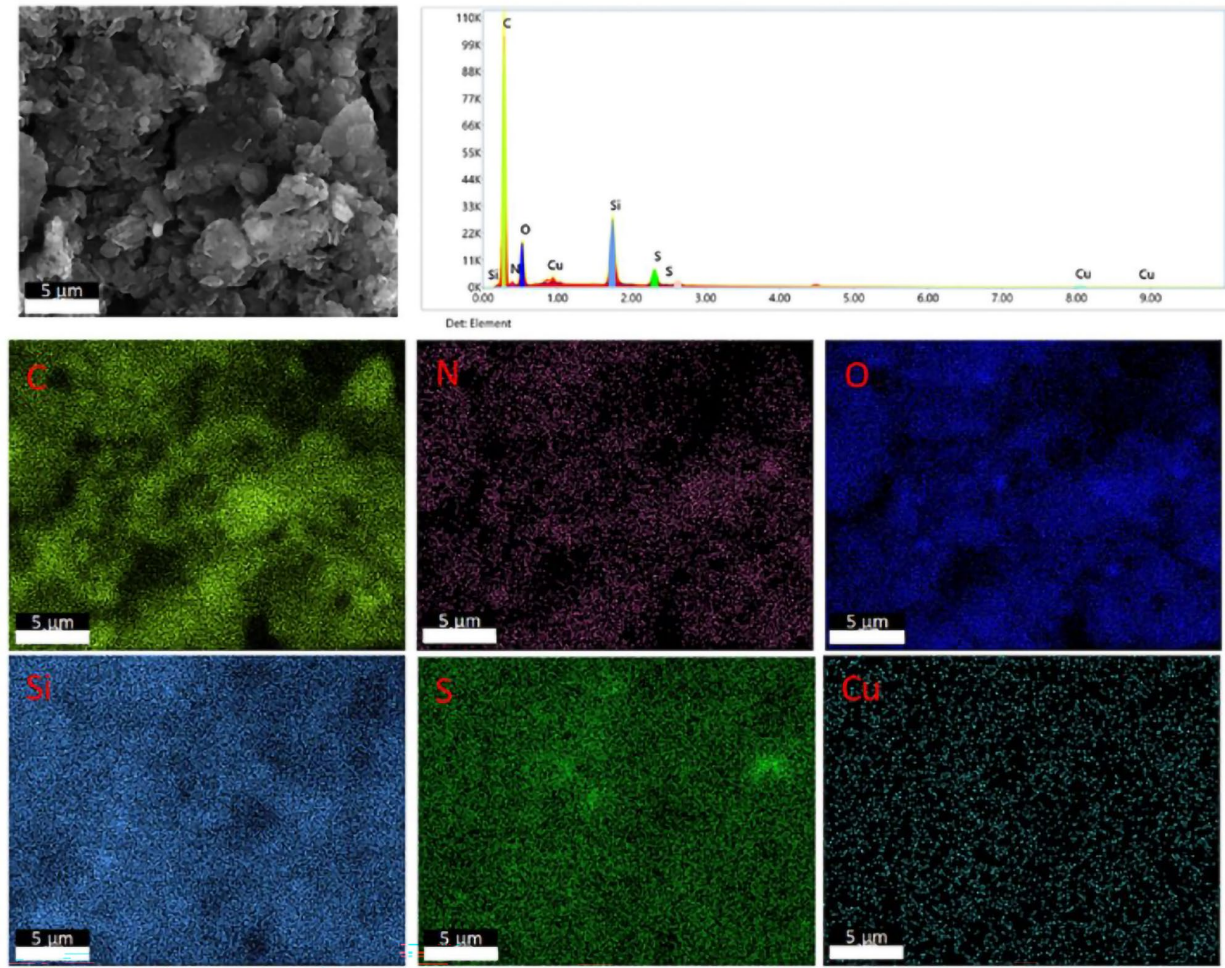
SEM–EDX analyses of Cu-rGO@PANI-DBSA.

Figures 11, 12 and 13 report the EDX analyses for Al_2_O_3_-rGO, steel-rGO, and Cu-rGO, all of them coated with PANI-DBSA. The EDX maps highlight the presence of the individual elements and the homogeneity of the samples, demonstrating the correct functionalization of the particles with PANI-DBSA.

Figure 12 shows also the FT-IR spectrum of the steel-rGO@PANI-DBSA composite in which several vibrational bands can be observed: at 3418 cm^−1^, a vibrational band due to the stretching of the O–H bond of rGO; at around 2900 cm^−1^ and 2800 cm^−1^, two intense vibrational bands due to the stretching of the CH_2_ and CH_3_ groups, respectively; at 1658 cm^−1^, an intense band due to the vibration of the carbonic structure of graphene sheets; the bands at 1511 and 1408 cm^−1^ are attributable, respectively, to the C = N and C = C stretching of the quinonoid and benzenoid units of PANI; the bands at 1300 and 1269 cm^−1^ are related to the C–N stretching of the PANI ring^64^; a weak vibrational band, at 1218 cm^-1^, originates from the vibration of the C–O–C group; weaker vibrational bands are observed in the range 1000–1200 cm^−1^, suggesting the presence of bonds such as C–H or C–O; a vibrational band at 668 cm^−1^ is attributable to the stretching of the S = O bond of the DBSA molecule. Overall, all these FT-IR observations clearly indicate the coating of steel particles with rGO and PANI-DBSA, in agreement with SEM/EDX analysis.

### Characterization of Al_2_O_3_-rGO@PANI-DBSA-PLA, steel-rGO@PANI-DBSA-PLA and Cu-rGO@PANI-DBSA-PLA composites

**Figure 14:**
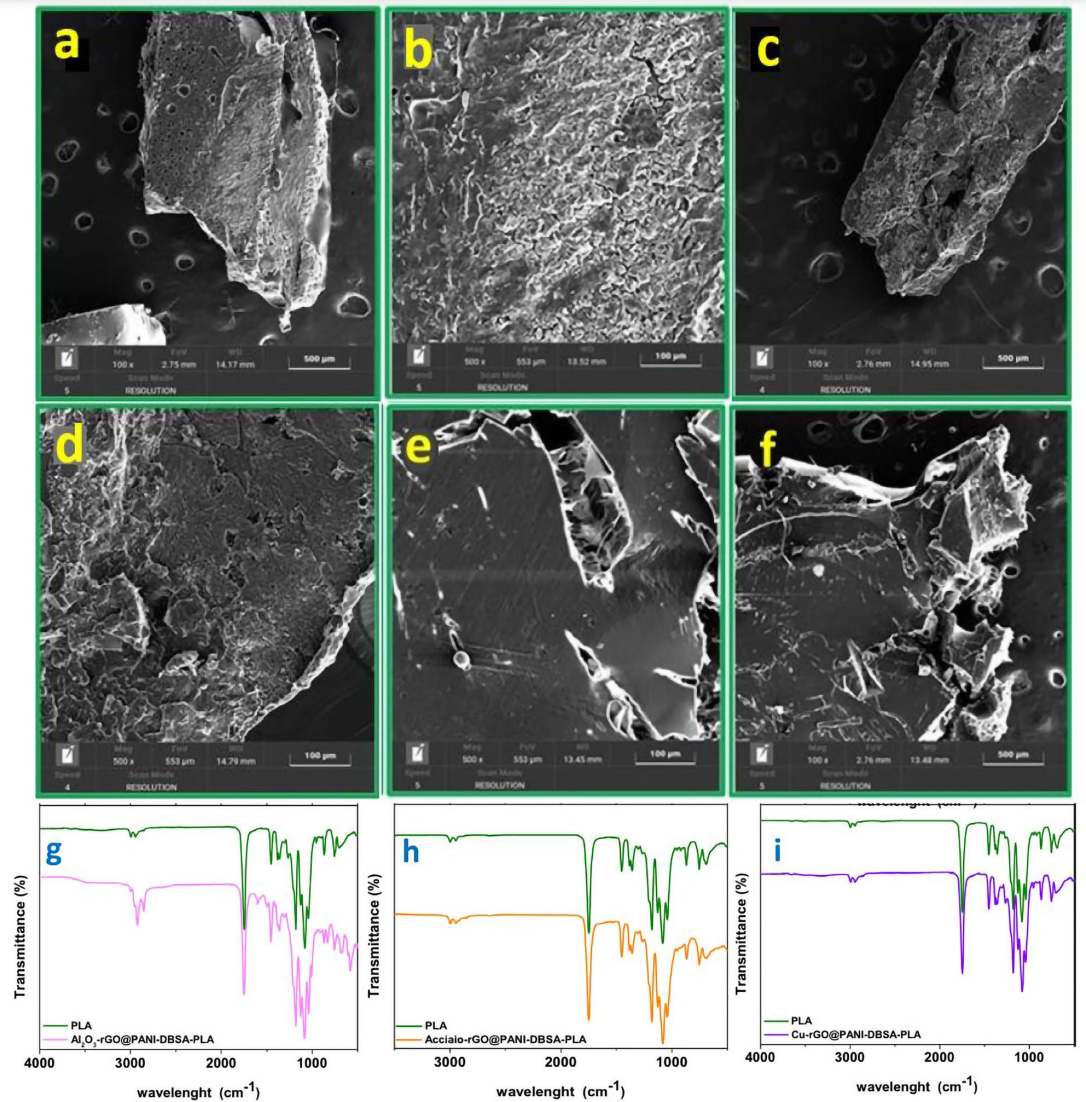
SEM images of: (**a**,**b**) Al2O3-rGO@PANI-DBSA-PLA scale bars: 500 and 100 µm; (**c**,**d**) steel-rGO@PANI-DBSA-PLA, scale bars: 500 and 100 µm; (**e**,**f**) Cu-rGO@PANI-DBSA-PLA 100 and 500 µm. FT-IR spectra of: (**g**) Al2O3-rGO@PANI-DBSA-PLA; (**h**) steel-rGO@PANI-DBSA-PLA (b); (i) Cu-rGO@PANI-DBSA-PLA.

The morphology of the composites Al_2_O_3_-rGO@PANI-DBSA-PLA, steel-rGO@PANI-DBSA-PLA and Cu-rGO@PANI-DBSA-PLA was also explored by means of SEM analyses (Fig. 14a–f). SEM images of all the samples show a rough surface with cracks and ridges.

FT-IR analysis was performed on the conductive composite Al_2_O_3_-rGO@PANI-DBSA-PLA (Fig. 14g), confirming the presence of PLA, with the vibrational bands of its CH, CH_2_ and CH_3_ bonds. In Fig. 14h, the FT-IR profile of steel-rGO@PANI-DBSA-PLA has also been reported. As can be seen, the bands of PLA (green profile in the same figure) are very intense and tend to mask the bands of the steel-rGO@PANI-DBSA sample. The bands in the range 2845–2990 cm^−1^ can be attributed to the stretching of the CH, CH_2_ and CH_3_ groups characteristic of PLA, PANI-DBSA, rGO and APTES as well, whereas the vibrational band characteristic of graphene sheets, at 1655 cm^−1^, is hidden by the band at 1750 cm^−1^ relative to the C = O stretching of PLA. Furthermore, at 1187 cm^−1^, a vibrational band due to the C–O–C stretching of PLA can also be detected. Moreover, a slight shift of the PANI band from 1466 cm^−1^ to 1447 cm^−1^ can be observed, probably due to interaction with PLA. The same phenomenon can be observed by looking at the band at 1216 cm^−1^, characteristic of the vibration of the C–O–C group of the rGO, which has moved to 1213 cm^−1^. Moreover, the vibrational band at 746 cm^−1^ related to the stretching of the S = O bond of the DBSA molecule is also slightly visible. Finally, the bands between 1000–500 cm^−1^ are due to the vibrations of the –OH, C–C, C-COO and C = bonds of the PLA^65^. The most evident bands are visible at 1750 cm^−1^ and at 1599 cm^−1^, due, respectively, to the stretching of the C = O bond of PLA and to the vibrations of the C = N bond of PANI. Eventually, Fig. 14i shows Cu-rGO@PANI-DBSA-PLA, which successfully confirms the occurred blending with PLA as well.

**Figure 15:**
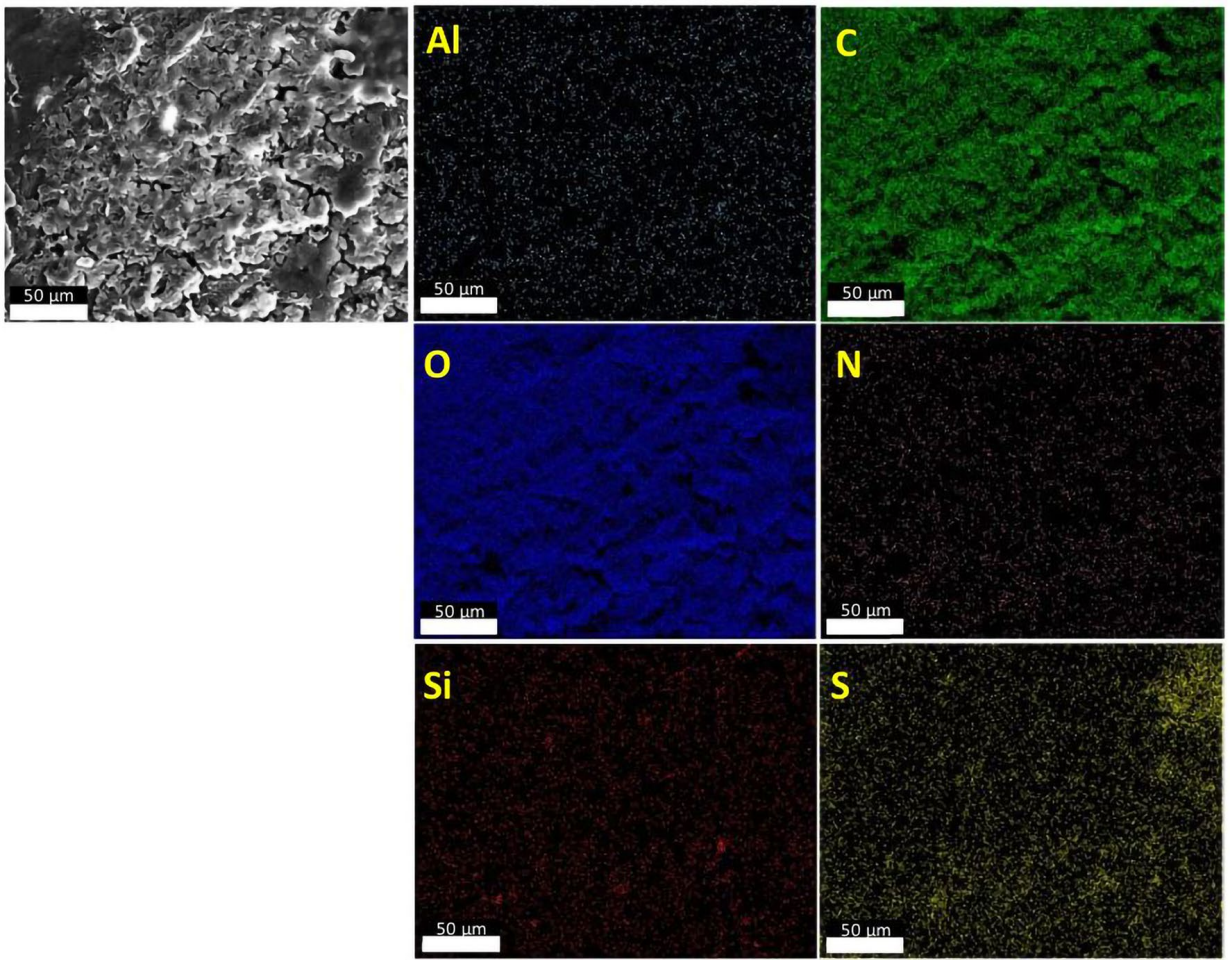
EDX maps of Al2O3-rGO@PANI-DBSA-PLA.

**Figure 16:**
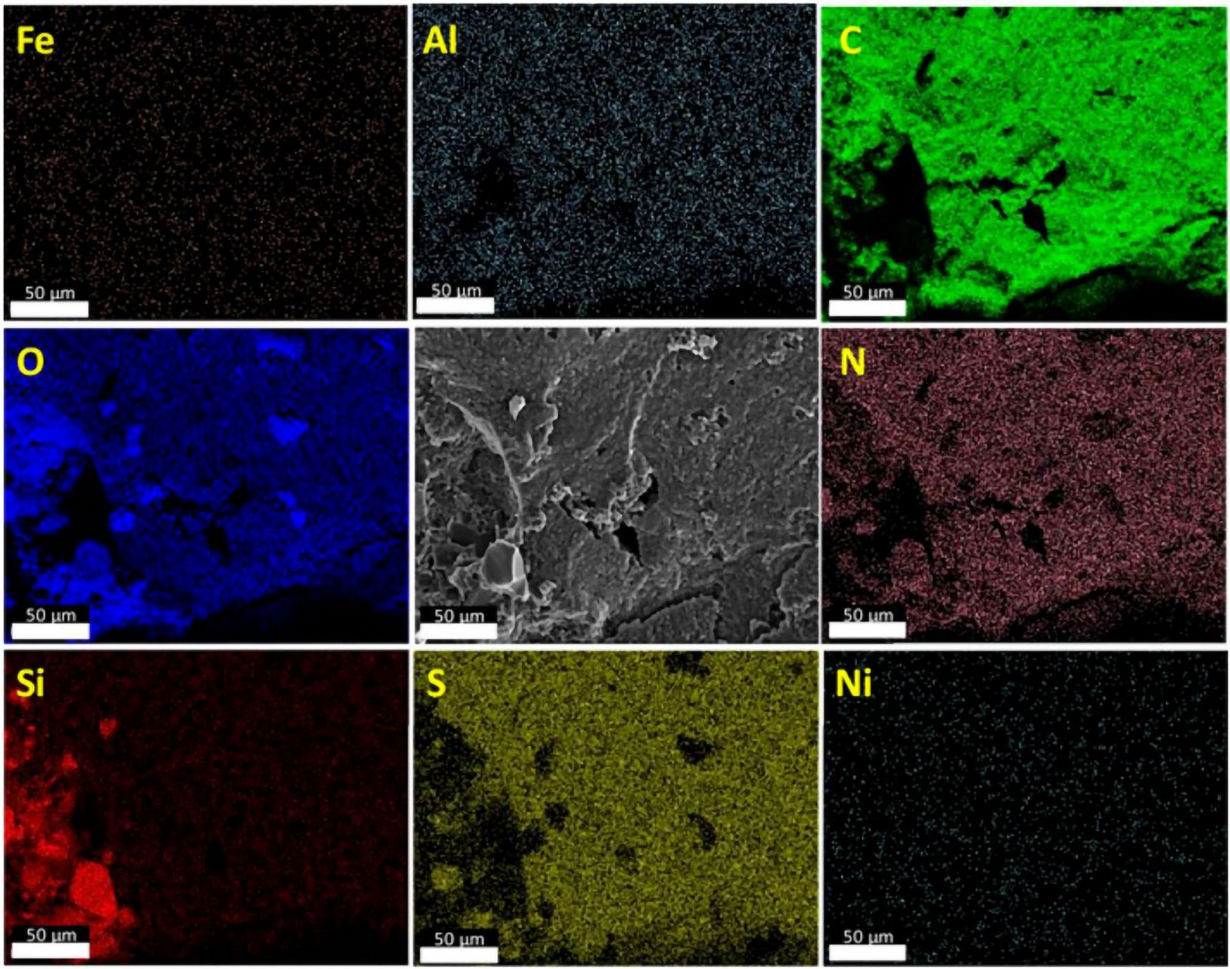
EDX maps of steel-rGO@PANI-DBSA-PLA.

**Figure 17:**
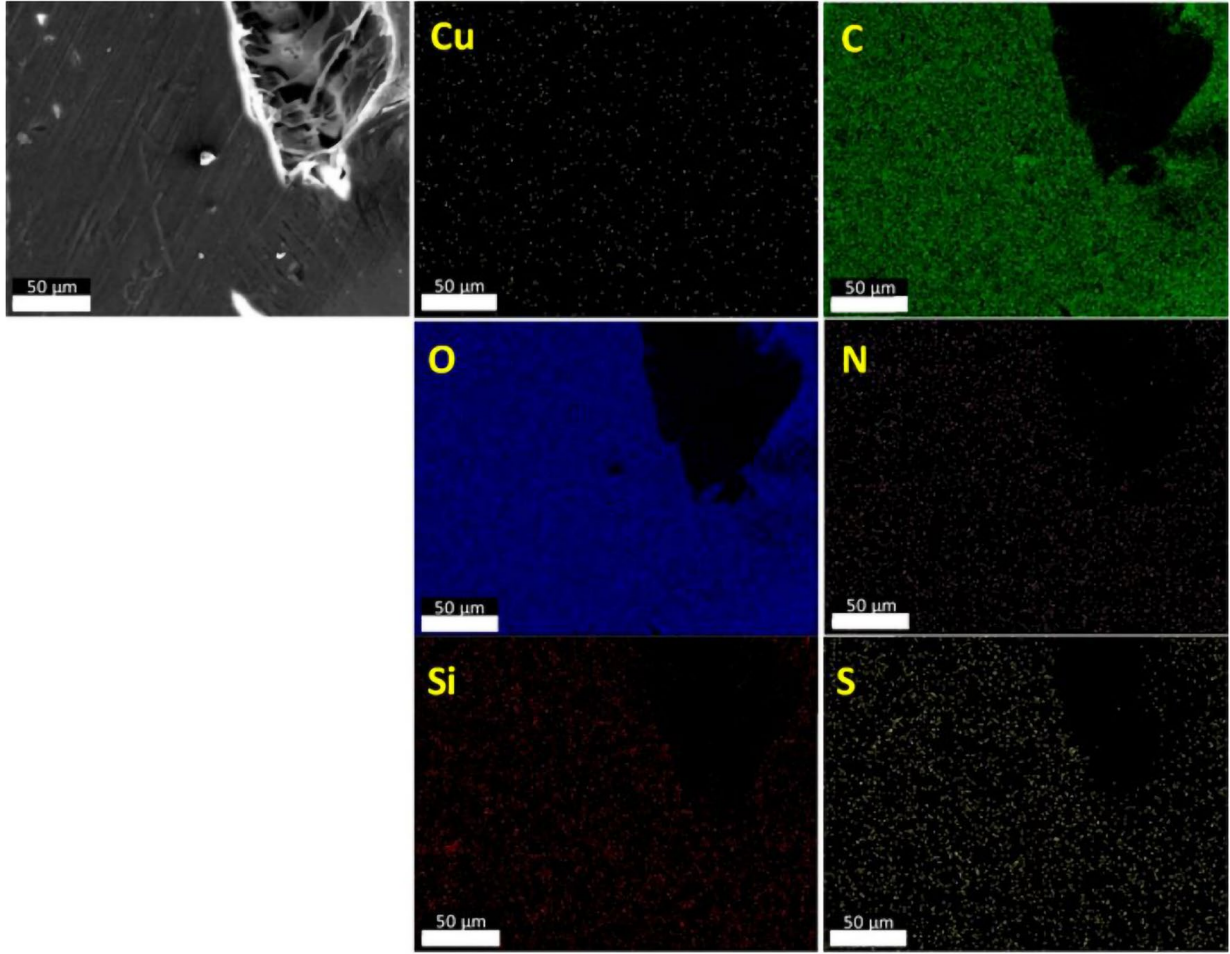
EDX maps of Cu-rGO@PANI-DBSA-PLA.

Figure 15 shows an SEM image (scale bar: 50 µm) and the corresponding EDX maps of Al_2_O_3_-rGO@PANI-DBSA-PLA. As can be seen, the maps confirm the presence of the composite, highlighting the occurrence of the following elements: carbon (C), oxygen (O), silicon (Si), nitrogen (N), sulfur (S), and aluminum (Al). Figure 16 and Fig. 17 show SEM images (scale bar: 50 µm) and the corresponding EDX maps of steel-rGO@PANI-DBSA-PLA and Cu-rGO@PANI-DBSA-PLA, respectively. The analyses highlight the uniform distribution of the elements constituting the composite: carbon (C), oxygen (O), silicon (Si), nitrogen (N), sulfur (S), nickel (Ni), iron (Fe) aluminum (Al) and copper (Cu).

### Electrochemical characterization of devices for supercapacitor applications

CV measurements recorded on the composites before being mixed with PLA were reported in Figures S6, S7 and S8, along with the profiles of the pristine microparticles. An increased charge accumulation compared to microparticles alone, likely due to graphene, was observed.

**Figure 18:**
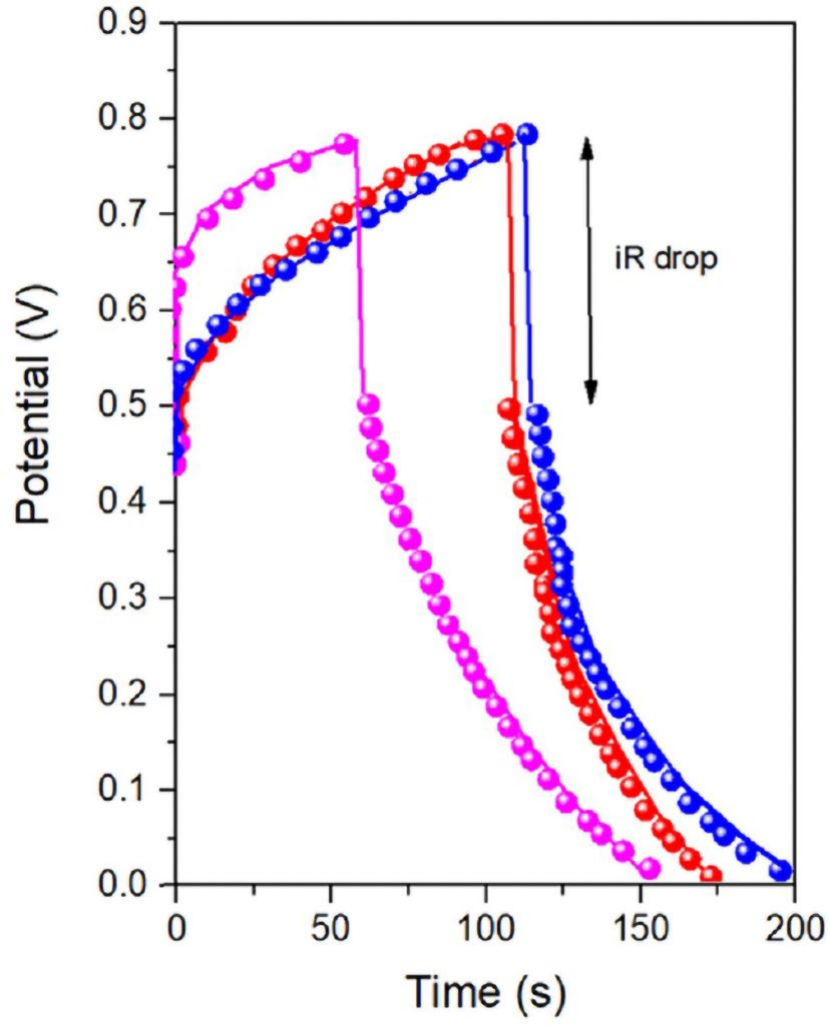
Galvanostatic charge–discharge curves of the devices at 1 A/g: (pink) Al2O3-rGO@PANI-DBSA-PLA, (red) steel-rGO@PANI-DBSA-PLA and (blue) Cu-rGO@PANI-DBSA- PLA.

**Figure 19:**
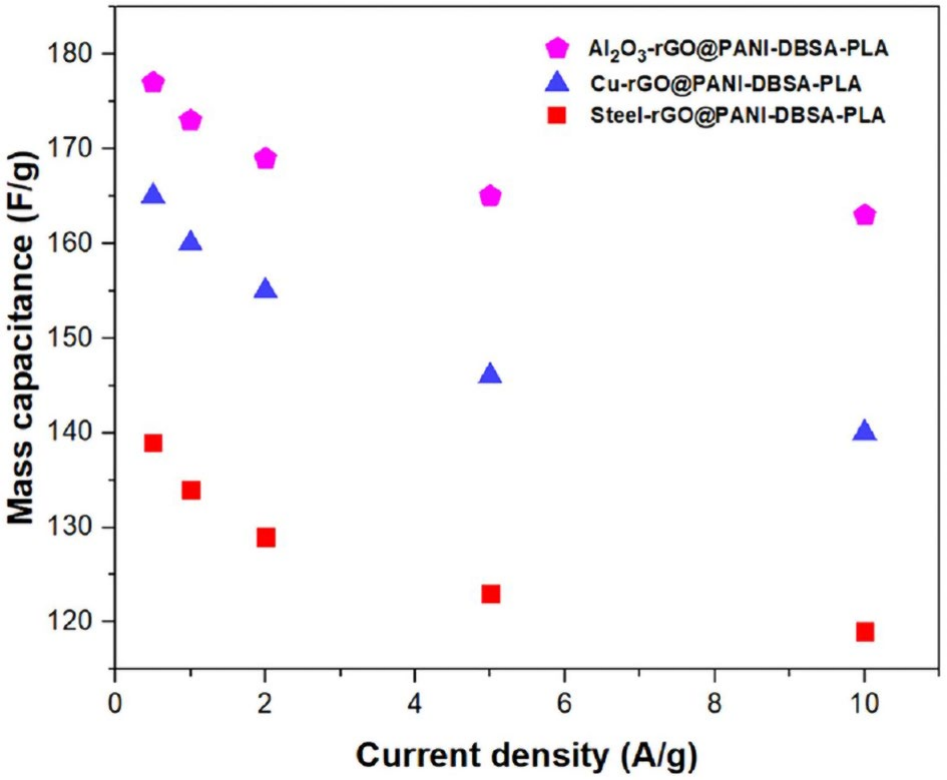
Specific capacitance vs. current density for: (pink) Al2O3-rGO@PANI-DBSA-PLA, (red) steel-rGO@PANI-DBSA- PLA and (blue) Cu-rGO@PANI-DBSA-PLA.

Moreover, to evaluate the capacitance performance of the final devices, GCD measurements were performed, with the PVA-H_2_SO_4_ acting as a solid-state electrolytic layer. Figure 18 shows the GCD curves, obtained at 1 A/g of Al_2_O_3_-rGO@PANI-DBSA-PLA (pink curve), steel-rGO@PANI-DBSA-PLA (red curve) and Cu-rGO@PANI-DBSA-PLA (blue curve). The three samples exhibit a stable performance, as can be seen from the similar size of the iR drop for each charge/discharge curve, and short discharge time. From them, specific capacitance values were calculated, according to Eq. (1): 173, 134 and 160 F/g for, Al_2_O_3_-rGO@PANI-DBSA-PLA, steel-rGO@PANI-DBSA-PLA and Cu-rGO@PANI-DBSA-PLA respectively. The capacitance values, in any case, higher than 100 F/g, are of similar magnitudes among the three samples, indicating the effectiveness of the approach. On the other hand, the observed differences can be attributed to the different particle size distributions, which, in turn, affect the amount of reduced graphene oxide assembled and the active surface areas available for electrochemistry. Additionally, the effective ion wettability of the samples, which depends on accessibility between particles, favored in the case of larger particles (see the small capacitance reduction for the Al_2_O_3_-support-based capacitors in Fig. 19) plays a crucial role. This effect is dominant even over the electrical conductivity of the materials, contributing the most to these differences. The above-mentioned Fig. 19 reports the mass capacitance values at different current densities for the three samples. The specific capacitance, as expected, decreased at increasing current densities in the range of 0.5–10 A/g, reaching 163, 119 and 140 F/g at 10 A/g for, Al_2_O_3_-rGO@PANI-DBSA-PLA, steel-rGO@PANI-DBSA-PLA and Cu-rGO@PANI-DBSA-PLA, respectively. This is an expected behavior since higher current densities correspond to shorter intervals for electrolyte ions to diffuse into the electrode channels and, therefore, they can access a smaller portion of the active material's surface area. Conversely, as previously observed, the electrical conductivity of the supports appears less relevant. Indeed, copper which is the most conductive and enjoys the higher rGO content and surface area, exhibits a capacitance of the same order of magnitude.

**Figure 20:**
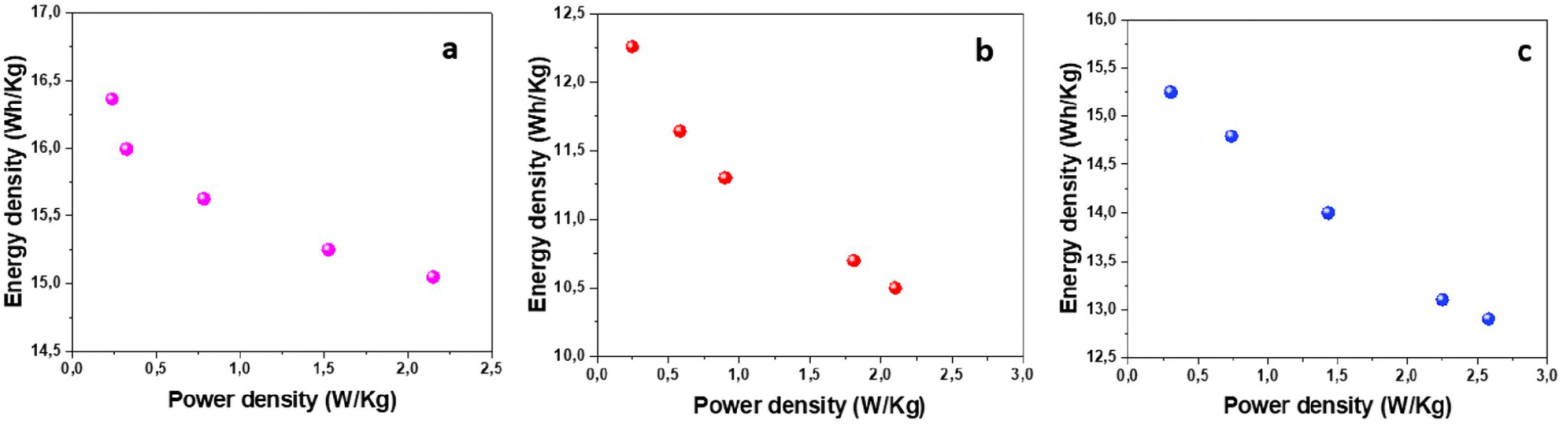
Ragone plot for: (**a**) Al2O3-rGO@PANI-DBSA-PLA, (**b**) steel-rGO@PANI-DBSA- PLA and (c) Cu-rGO@PANI-DBSA-PLA.

Starting from the specific capacitance values, energy densities and power densities of the devices were also evaluated according to (2) and (3) (see Ragone plots in Fig. 20). Values of energy and power densities were recorded, respectively, in the range of 10.5–15 Wh/kg and 2.21–2.58 W/kg.

**Figure 21:**
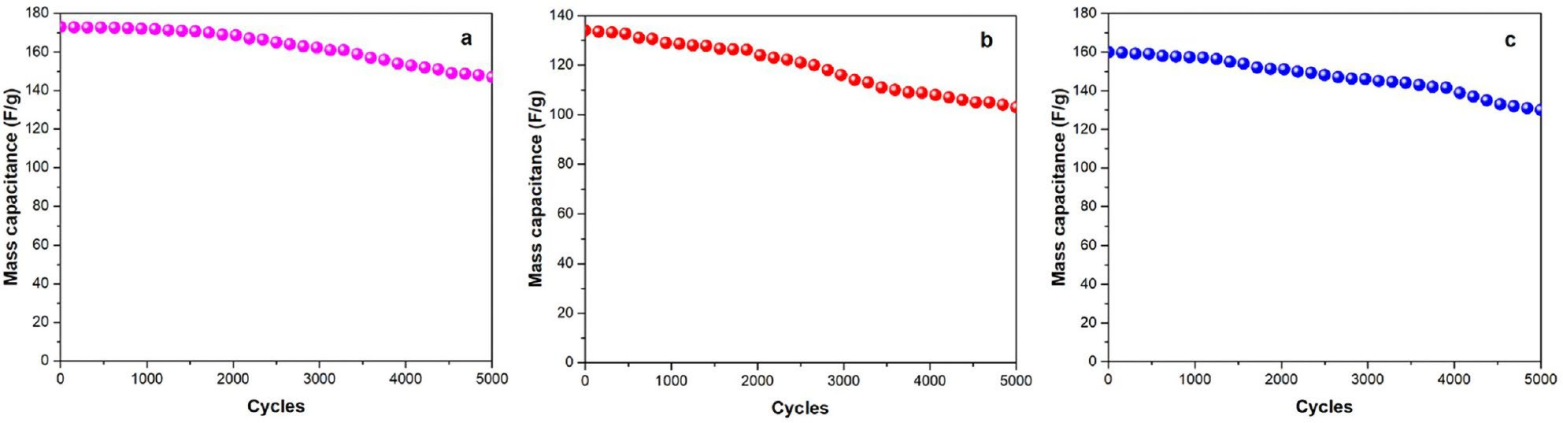
Capacitance vs. number of cycles at 1 A/g for: (**a**) Al2O3-rGO@PANI-DBSA-PLA, (**b**) steel-rGO@PANI-DBSA-PLA and (c) Cu-rGO@PANI-DBSA-PLA.

Furthermore, the as-obtained supercapacitors were tested after several cycles of usage, showing encouraging results in terms of durability. In particular, as reported in Fig. 21, Al_2_O_3_-rGO@PANI-DBSA-PLA, steel-rGO@PANI-DBSA-PLA and Cu-rGO@PANI-DBSA-PLA retain 85%, 77%, and 81% of their initial capacitance values after 5000 cycles, respectively.

These values, along with the specific capacitances and the energy densities, prove that the obtained results are comparable with those reached with non-3D-printed composites including rGO and other graphene-based materials, even when comparing the ones with finer sizes (see Table 1). The as-obtained results are likely due to the enhanced stability of the GO self-assembling 3D structures on surface-functionalized robust materials, as well as the combination in the composite structure of rGO and PANI. The former guarantees good wettability with the electrolyte prevents aggregations of adjacent graphene sheets, and contributes to the capacitive behavior of the system due to its high surface area and conductive sheets. The latter guarantees enhanced electrical conductivity while also contributing to the overall capacitance^66^.

**Table 1:** Supercapacitive performance of the 3D printed devices and some of the most performing non-3D-printed composites including rGO and other graphene-based materials in literature.

Graphene-based materials for supercapacitor applications (with material size when cited)	Specific capacitance [F/g_material_]	Capacitance retention vs. cycles	Energy density [Wh/kg]	Refs.
Cu_2_O/CuO NPs (25 nm, 5 nm) on rGO nanosheets	136.3 (at 10 A/g)	78.6% of its initial capacitance after 10,000 cycles at 10 A/g	−	^67^
Chemically converted graphene/stainless-steel fabric (40 μm)	45.1 F/g (at 0.25 A/g)	96.8% of the initial capacitance after 7500 cycles at 8 mA/cm^2^	19.2	^68^
rGO hydrogel/stainless steel mesh	147.2 (at 10 A/g)	93.1% of the initial capacitance after 10,000 cycles at 2 A/g	−	^69^
rGO-Fe_3_O_4_ NPs (14.1 nm) -NH-PANI	132.9 (at 1 mA/cm^2^)	about 94% of the initial capacitance after 2000 cycles at 10 mA/cm^2^	11.8	^70^
rGO nanosheets-AuNPs (5–20 nm) @PANI (two-electrode configuration)	193.8 (at 5 A/g)	86.9% of the initial capacitance after 5000 cycles at 2 A/g	6.72	^71^
Graphene-Cu_2_O nanocomposite/Cu foil (solid-state device)	11.94 (at 10 mV/s)	-	6.63	^72^
rGO/Pin (71 nm)/Al_2_O_3_ (59 nm) nanocomposite (solid-state device)	38.46 (at 5 A/g)	83% of its initial capacitance after 5000 cycles	10	^73^
Al_2_O_3_-rGO@PANI-DBSA-PLA (solid-state device)	163 (at 10 A/g)	85% of its initial capacitance after 5000 cycles	15	This work
steel-rGO@PANI-DBSA-PLA (solid-state device)	119 (at 10 A/g)	77% of its initial capacitance after 5000 cycles	10.5	This work
Cu-rGO@PANI-DBSA-PLA (solid-state device)	140 (at 10 A/g)	81% of its initial capacitance after 5000 cycles	13	This work

Therefore, the obtained results suggested the successful manufacturing through AM of the above-prepared materials and highlighted the possibility of easily and economically creating energy storage devices made up of 3D printable materials with good performance. As for future perspectives, future improvements in performance can be achieved, for instance, by adjusting the percentage of electrochemically active material in the composites.

## Conclusion

In this study, with the aim of investigating a versatile and adaptable approach for obtaining robust and performing 3D printed supercapacitors, various architectures, that benefit from graphene covering, were reported and studied. In particular, Al_2_O_3_-, steel-, and Cu-based microparticles have been explored for the realization of 3D self-assembling materials covered with rGO to be processed through AM.

In detail, the manufacturing procedure of the devices can be summarized as follows: (i) the surface of the particles of Cu, Al_2_O_3_ and steel was first functionalized with amino groups and then covered through self-assembly with GO, which covered the particles in the form of reduced rGO; (ii) a PANI-DBSA complex was created; (iii) to further improve the conductivity and processability characteristics for 3D printing, a self-assembly process was carried out between the particles coated with carbonaceous material and the solution containing the PANI-DBSA complex; (IV) the as-obtained composites were mixed with an optimum amount of PLA in order to further improve AM processability of the composites; (V) the PLA containing-composites were extruded in filaments, which were then printed through FDM technique to create circular disc electrodes which were, eventually, assembled in solid-state electrolyte-based symmetric devices. After a broad characterization of both the pristine particles and the final PLA-containing composites through FT-IR, TG-DTG, SEM and EDX techniques, which allowed to confirm their nature, the devices were tested in terms of energy storage through GCD tests.

Capacitance values, in the order of hundreds of F/g, are of similar magnitudes among the three samples, indicating the effectiveness of the approach. On the other hand, the observed small differences can be attributed to the different particle size distributions, which, in turn, affect the amount of reduced graphene oxide assembled and the active surface areas available for electrochemical reactions. Additionally, the effective ion wettability of the samples, which depends on accessibility between particles in the pathway towards the active surface, favored in the case of larger particles, plays a crucial role in these differences. Conversely, the electrical conductivity of the supports seems less relevant. Indeed, copper which is the most conductive and benefits from the higher rGO content and surface area, exhibits capacitance of a similar order of magnitude.

The energy density values and good durability demonstrate that the results obtained are comparable to those achieved with non-3D-printed, high-performance composites containing rGO and other graphene-based materials, even when considering materials with finer sizes. This is likely due to the advantage of the inclusion of high-performance rGO and PANI in self-assembling 3D structures based on surface-functionalized very robust materials. Therefore, the obtained results suggested the successful manufacturing through AM of the above-prepared materials and highlighted the possibility of easily and economically creating energy storage devices made up of 3D printable materials with good performance. As for future perspectives, better performance can be reached, for instance, by modifying the percentage of electrochemically active material in the composites. These results they also open the way for the creation of more complex structures high surface area structures.

### Supplementary Information

Supplementary Information.

## Data Availability

The datasets used and/or analysed during the current study are available from the corresponding author on reasonable request.
