# Supplementary material for: Self-assembled monolayers of reduced graphene oxide for robust 3D-printed supercapacitors
Source: Sci Rep. 2024 Jul 1;14:14998. doi: 10.1038/s41598-024-65635-8 (PMC11217268; doi:10.1038/s41598-024-65635-8)
Supplement: Supplementary file 1 — Supplementary Information. [file 41598_2024_65635_MOESM1_ESM.docx]

**Supporting Information**

**Characterization of Al_2_O_3_-rGO, steel-rGO, and Cu-rGO composites**

Regarding the characterization of the products obtained after the first two steps, the XRD spectra of the rGO-covered functionalized Cu and Al_2_O_3,_ named Al_2_O_3_-rGO and Cu-rGO, are shown in Figure S1. The presence of the characteristic peaks of the crystalline phases of α-Al_2_O_3_ (green profile) and of metallic Cu (blue profile), can be observed. In both spectra, a peak at around 25 ° is also clearly visible, which can be attributed to the (002) plane of graphene, hence being indicative of the occurred reduction of graphene oxide to rGO. By means of this analysis, therefore, not only the correct functionalization of the particles was demonstrated, but also the occurred reduction of graphene oxide to rGO, probably due to its anchoring to the active sites of the amino-functionalized particles.


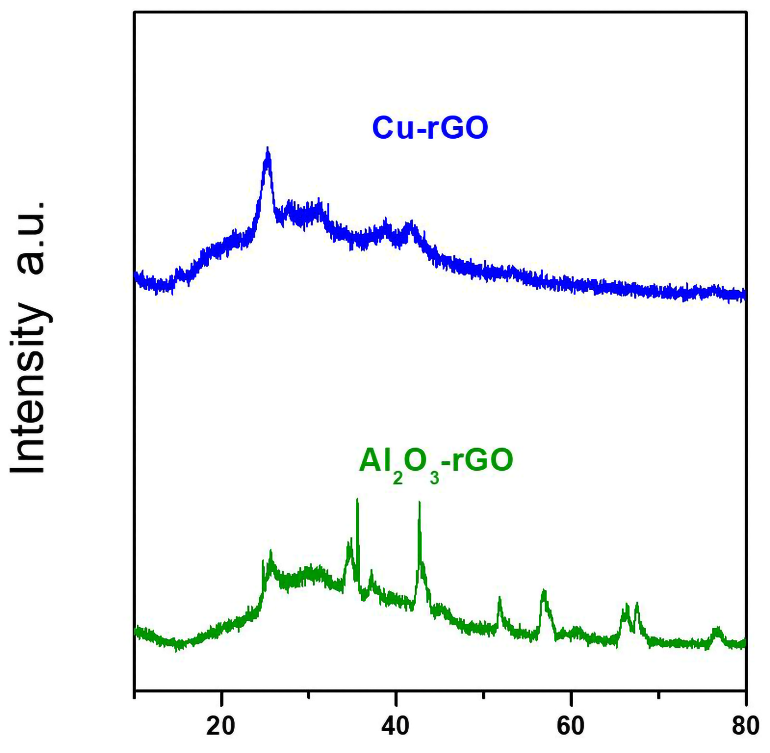


***Figure S1. X-ray diffraction patterns of Al_2_O_3_-rGO (green profile) and Cu-rGO (blue profile)***

As for the X-ray diffraction spectrum of the Cu-rGO sample, peaks at 28.7 °, 32.1 °, 39.8 ° are also visible, corresponding, respectively, to the (112), (110) and (111) typical crystal planes of CuO^1^

The FT-IR spectrum of the Al_2_O_3_-rGO sample is shown in Figure S2a. From the analysis of the spectrum, the occurred reduction of graphene oxide to rGO can be confirmed since the typical GO absorbance bands of the O–H, C=O and C–O groups cannot be detected^2^. The spectrum shows a vibrational band at 533 cm^-1^ due to the vibration of the Si–O–Si bond of the APTES molecule. Furthermore, the intensity of the bands related to the O–Al–O bond is reduced, probably due to the coating with rGO. Figure S2b shows the FT-IR spectrum of the Cu-rGO sample. The spectrum confirms the occurred reduction of graphene oxide since, even in this sample, the typical GO absorbance bands of the O–H, C=O, and C–O groups cannot be detected. The spectrum shows the slight symmetrical and asymmetrical vibrational bands of the CH_2_ group of the APTES molecule at 2920 cm^-1^ and at 2847 cm^-1^ ^3^. Finally, an absorption band at 545 cm^-1^ can be observed, due to the vibration of the Cu–O bond of CuO^4^


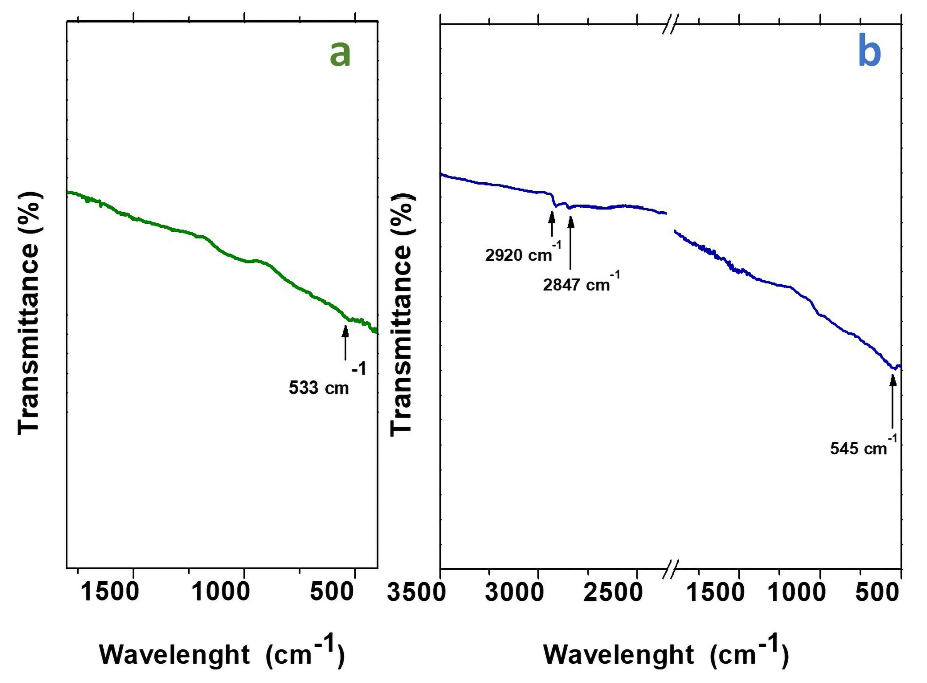


***Figure S2. FT-IR spectra of Al_2_O_3_-rGO (a) and Cu-rGO (b)***

The thermogravimetric analysis of the Al_2_O_3_-rGO sample (Figure S3) shows two distinct weight losses: an initial one, due to the functional groups containing residual oxygen and a second one, owing to the degradation of the rGO structure. Moreover, the thermogram of the Cu-rGO sample (Figure S4) shows a weight loss at around 350 °C, attributable to the degradation of the oxygenated functional groups. A further weight loss can be observed in the range of 400-650 °C, which corresponds to the degradation of the C−C bonds of rGO^5^.


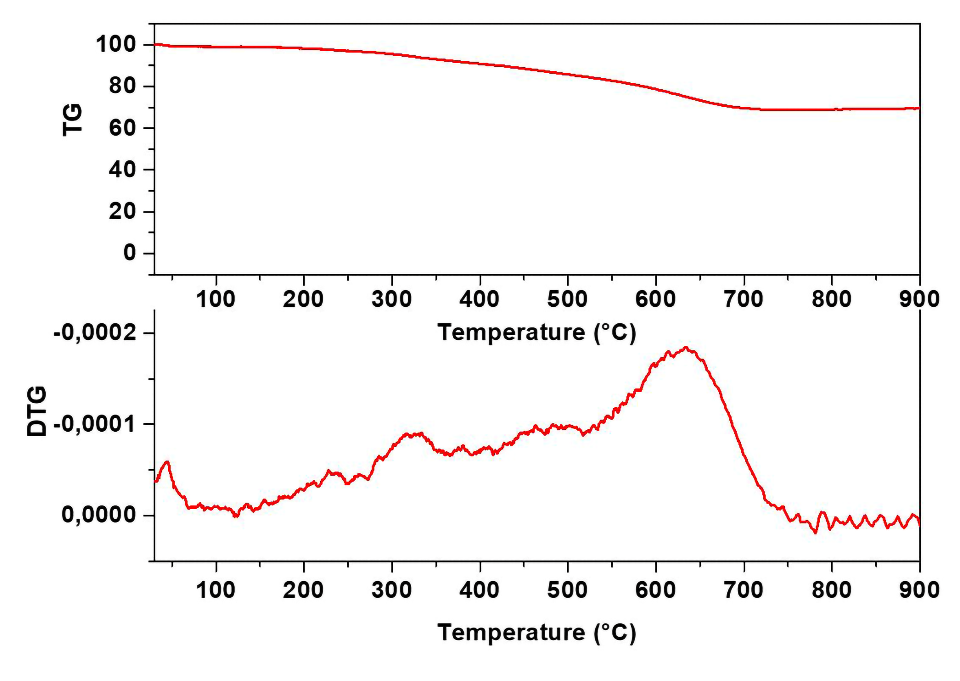


***Figure S3. TG-DTG resulting graphs of Al_2_O_3_-rGO***


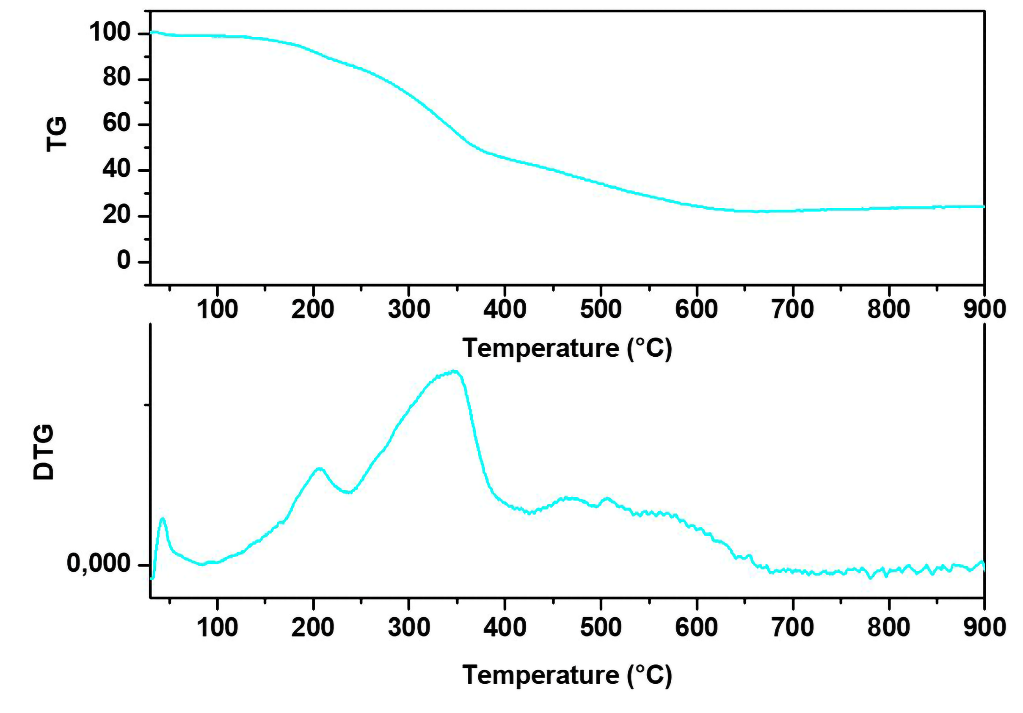


***Figure S4.*** ***TG-DTG resulting graphs of Cu-rGO***

Steel particles were also functionalized with amino groups and coated with rGO. As with the other powders, FT-IR and TG-DTG analyses demonstrated successful functionalization.

**Thermogravimetic analysis of the steel-rGO@PANI-DBSA**

Moreover, the thermogravimetic analysis of the steel-rGO@PANI-DBSA sample, see Figure S5, shows a substantial weight loss due to water and other solvents adopted during synthesis, showing a residue of 12 wt.%.

**
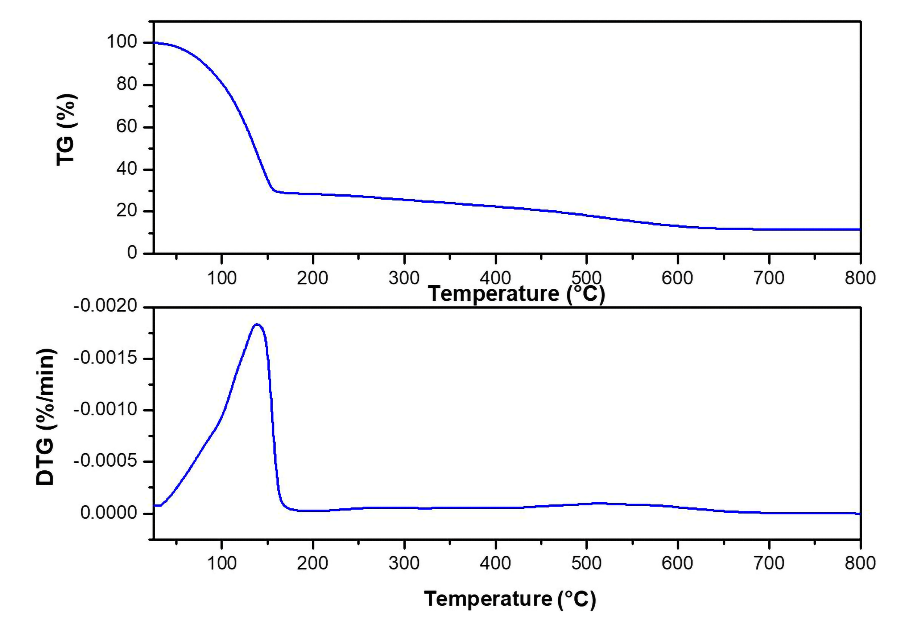
**

***Figure S5. TG-DTG results of the*** ***steel-rGO@PANI-DBSA sample***

**Thermogravimetric analysis of the composites with PLA**

The TG-DTG graphs of Al_2_O_3_-rGO@PANI-DBSA-PLA, steel-rGO@PANI-DBSA-PLA, and Cu-rGO@PANI-DBSA-PLA, presented in Figures S6, S7, and S8 respectively, show significant weight loss around 370°C, which starts at lower temperature for Al_2_O_3_-rGO@PANI-DBSA-PLA probably due to the acidic behavior of the alumina surfaces, and a further smaller loss between 400°C and 650°C. While the latter corresponds to the degradation of the C−C bonds of rGO^5^, the former can be attributed to the decomposition of the organic chains, particularly PLA^6^, which, being in a weight ratio of 2:1 with the composite, is the prevalent material. The polymer matrix ensures the stability of the sample at extrusion temperatures, as corroborated by Figures S9, S10, and S11, confirming short-term thermal stability at 200°C, sufficient for extrusion. Specifically, the weights of steel-rGO@PANI-DBSA-PLA and Cu-rGO@PANI-DBSA-PLA remain constant for at least an hour under airflow, while Al_2_O_3_-rGO@PANI-DBSA-PLA shows a slight degradation, retaining 95.2% of its weight after 60 minutes.

***
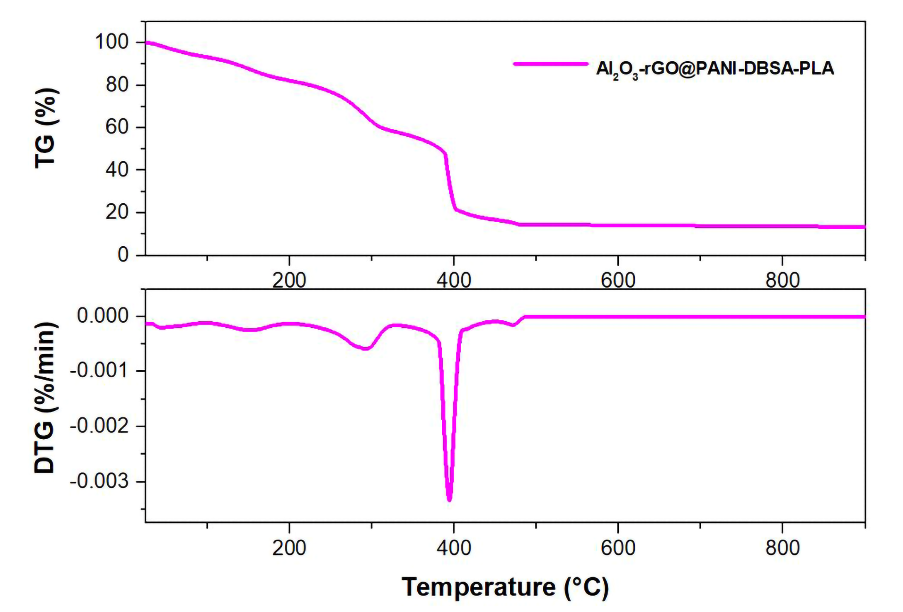
***

***Figure S6. TG-DTG resulting graphs of*** ***Al_2_O_3_-rGO@PANI-DBSA-PLA***

***
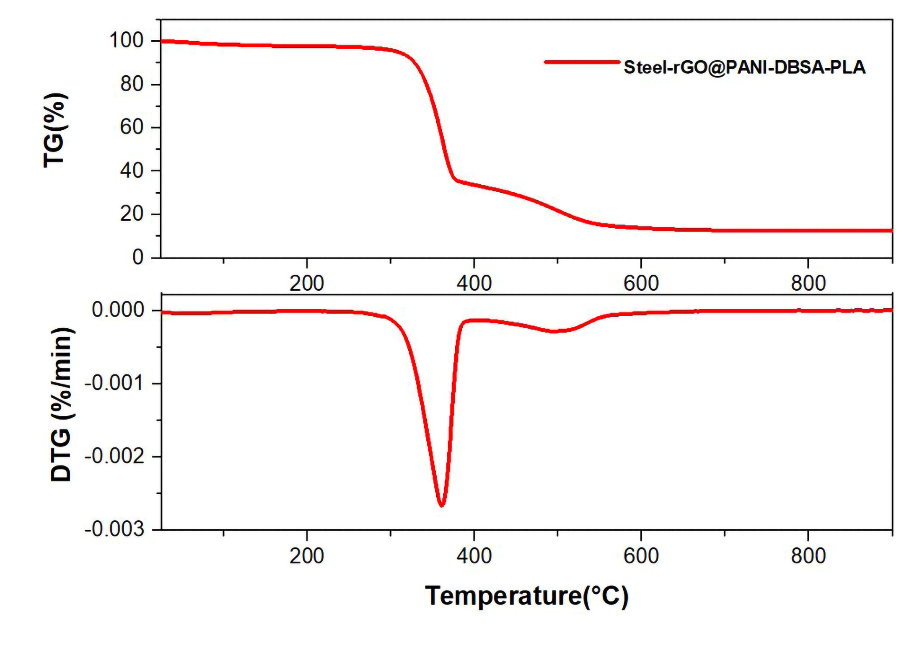
***

***Figure S7. TG-DTG resulting graphs*** ***of steel-rGO@PANI-DBSA-PLA***

***
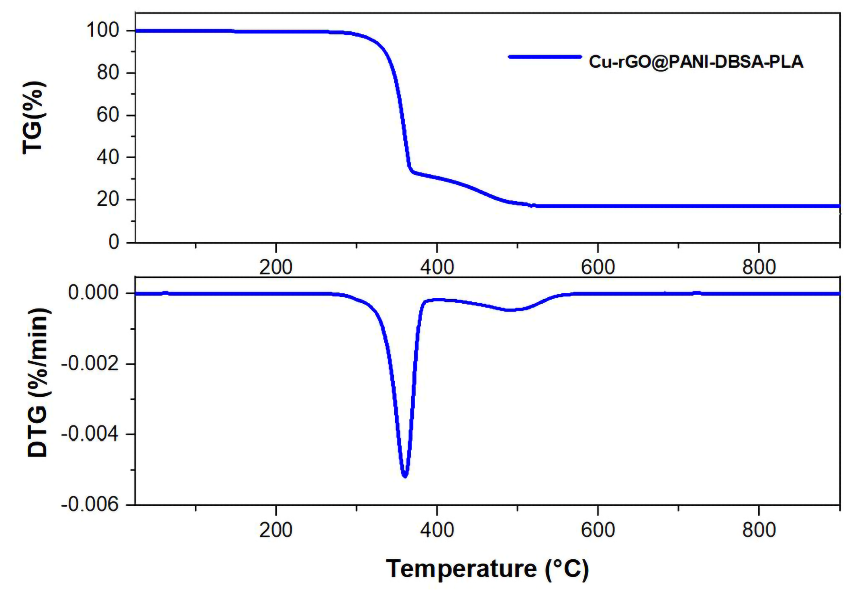
***

***Figure S8. TG-DTG resulting graphs of*** ***Cu-rGO******@PANI-DBSA-PLA***

***
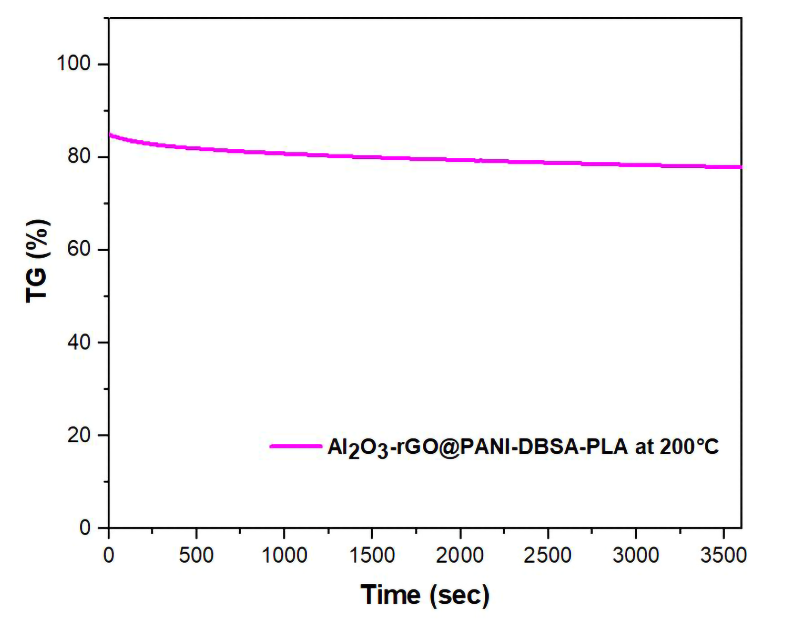
***

***Figure S9. TG of Al_2_O_3_-rGO@PANI-DBSA-PLA at 200°C for 60 min***

***
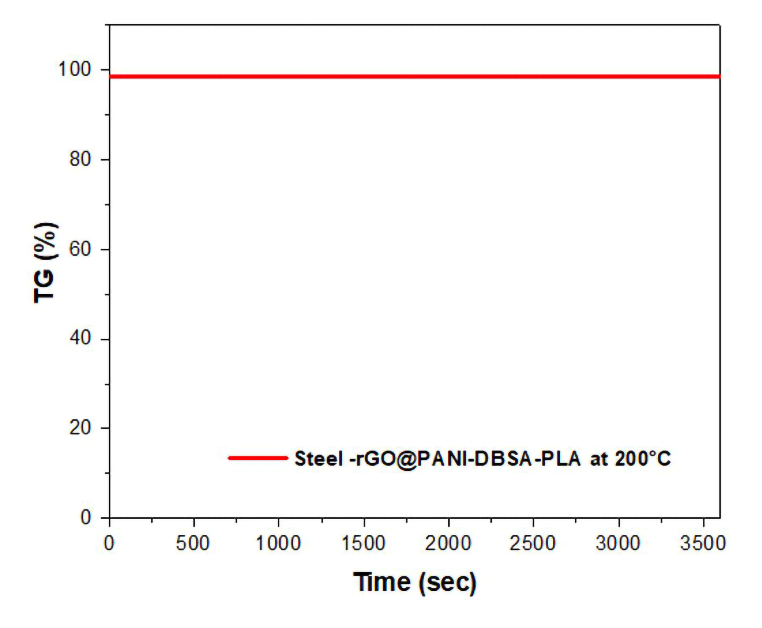
***

***Figure S10. TG of steel-rGO@PANI-DBSA-PLA at 200°C for 60 min***

***
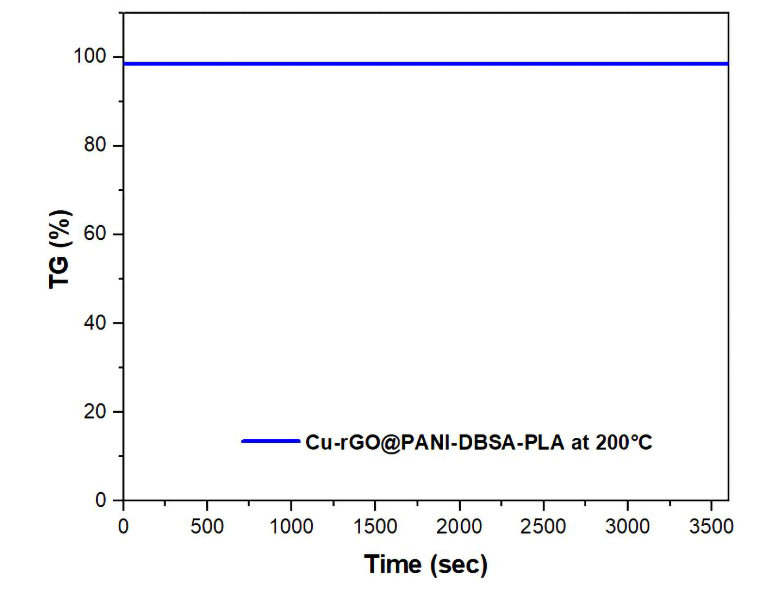
***

***Figure S11. TG of Cu-rGO@PANI-DBSA-PLA at 200°C for 60 min.***

**Electrochemical characterization of pristine microparticles, Al_2_O_3_-rGO@PANI-DBSA, steel-rGO@PANI-DBSA and Cu-rGO@PANI-DBSA for supercapacitor electrode applications**

Current–voltage measurements are crucial in determining the capacitance performance of the samples as supercapacitor electrodes. Therefore, cyclic voltammograms in the optimal voltage ranges of -0,2÷-1 V, −0,2÷-0,7 V and -0,2÷-0,8 V were recorded in a three-electrode-setup-based cell filled with a 0.5 M H_2_SO_4_ electrolytic solution to evaluate the electrochemical behavior of Al_2_O_3_-rGO@PANI-DBSA, steel-rGO@PANI-DBSA and Cu-rGO@PANI-DBSA, (see Figures S12, S13 and S14, respectively) at a scan rate of 20 mV/s. As can be seen from the figures, no detectable redox peaks can be identified in the chosen voltage range for all samples, both before and after functionalization. Therefore, the composite materials show quasi-rectangular current–voltage response curves, suggesting a nearly ideal capacitive behavior of the prepared samples ^7,8^. Moreover, the percentage increase in mass capacitance after functionalization is particularly relevant for the Cu (81%) and the steel particles (75%), while the difference between the Al_2_O_3_ particles and the Al_2_O_3_-rGO@PANI-DBSA composite is less (40%), probably since the amount of active rGO covering Al_2_O_3_ is reduced compared to the other composites, as highlighted by Figure S3.


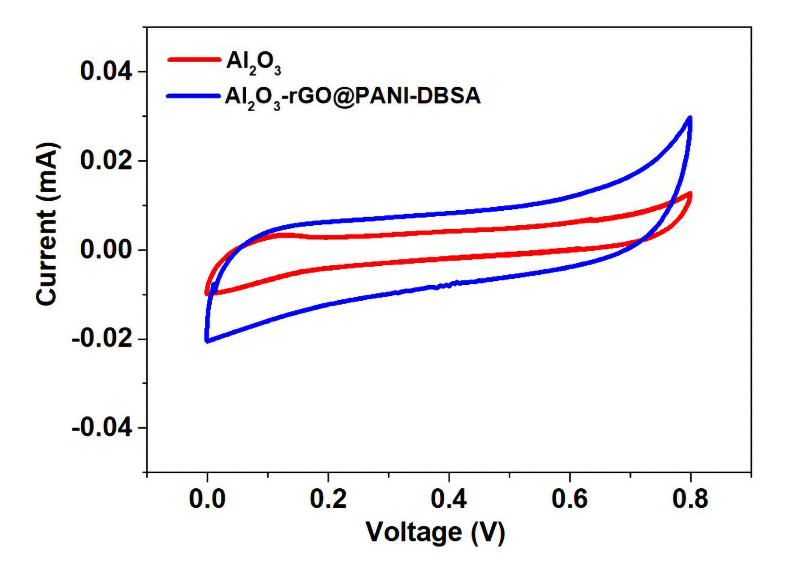


***Figure S12. Cyclic voltammograms of Al_2_O_3_ (red profile) and*** ***Al_2_O_3_-rGO@PANI-DBSA composite (blue profile) at 20 mV/sec***

***
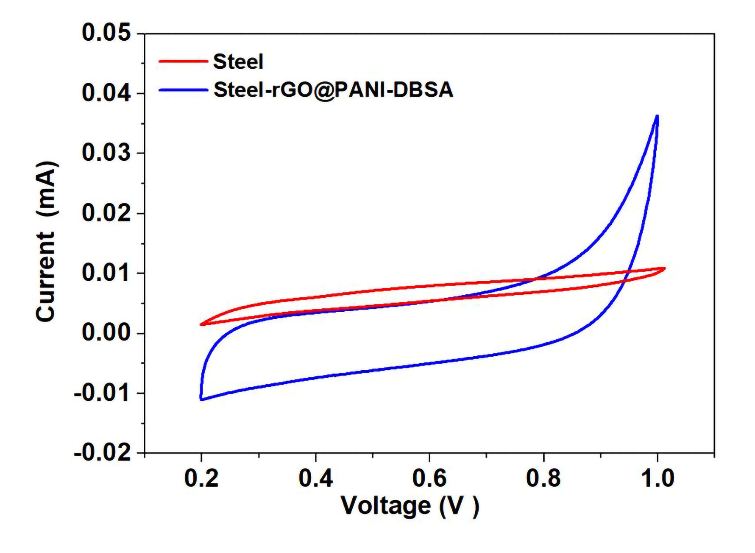
***

***Figure S13. Cyclic voltammograms of steel particles (red profile) and Steel-rGO@PANI-DBSA composite (blue profile) at 20 mV/sec***

*
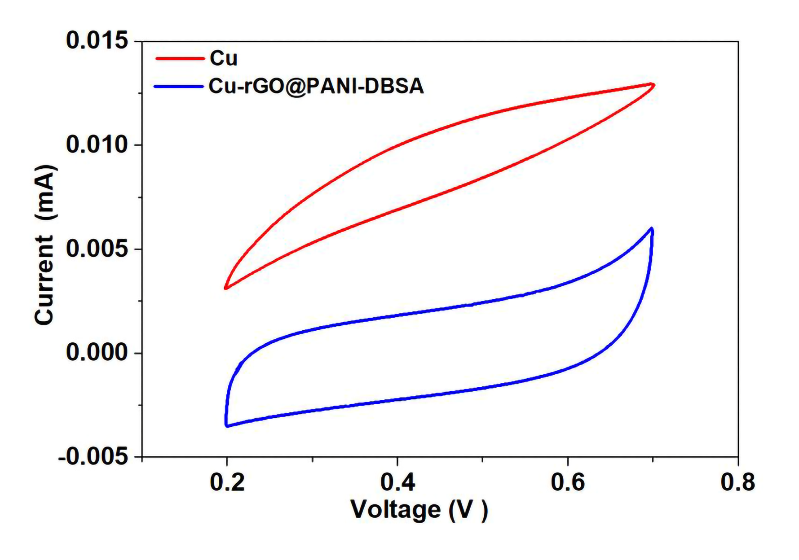
*

***Figure S14. Cyclic voltammograms of Cu particles (red profile) and Cu-rGO@PANI-DBSA composite (blue profile) at 20 mV/sec***

**References**

1. Shinde, S. K. *et al.* Influence of Mn incorporation on the supercapacitive properties of hybrid CuO/Cu(OH)2 electrodes. *RSC Adv* **5**, 30478–30484 (2015).

2. Maharsi, R., Arif, A. F., Ogi, T., Widiyandari, H. & Iskandar, F. Electrochemical properties of TiOX/rGO composite as an electrode for supercapacitors. *RSC Adv* **9**, 27896–27903 (2019).

3. Hu, Y. *et al.* Electrostatic self-assembly preparation of reduced graphene oxide-encapsulated alumina nanoparticles with enhanced mechanical properties of alumina nanocomposites. *J Eur Ceram Soc* **38**, 5122–5133 (2018).

4. Sundar, S., Venkatachalam, G. & Kwon, S. J. Biosynthesis of copper oxide (Cuo) nanowires and their use for the electrochemical sensing of dopamine. *Nanomaterials* **8**, (2018).

5. Gupta, A., Jamatia, R., Patil, R. A., Ma, Y. R. & Pal, A. K. Copper Oxide/Reduced Graphene Oxide Nanocomposite-Catalyzed Synthesis of Flavanones and Flavanones with Triazole Hybrid Molecules in One Pot: A Green and Sustainable Approach. *ACS Omega* **3**, 7288–7299 (2018).

6. Yan, Y. *et al.* Preparation and characterization of intumescent flame retardant biodegradable poly(lactic acid) nanocomposites based on sulfamic acid intercalated layered double hydroxides. *Fibers and Polymers* **18**, 2060–2069 (2017).

7. Wang, Z. & Liu, C. J. Preparation and application of iron oxide/graphene based composites for electrochemical energy storage and energy conversion devices: Current status and perspective. *Nano Energy* **11**, 277–293 (2015).

8. Sarno, M., Ponticorvo, E. & Scarpa, D. Ru and Os based new electrode for electrochemical flow supercapacitors. *Chemical Engineering Journal* **377**, 120050 (2019).
